# Using genetic instruments to estimate the causal effect of hormonal reproductive factors on osteoarthritis

**DOI:** 10.3389/fpubh.2022.941067

**Published:** 2022-11-14

**Authors:** Bingran Wang, Junhua Wu, Han Li, Xiaoyan Jin, Cong Sui, Zhen Yu

**Affiliations:** ^1^Department of Obstetrics and Gynecology, The First Affiliated Hospital of Anhui Medical University, Hefei, China; ^2^Anhui Province Key Laboratory of Reproductive Health and Genetics, Anhui Medical University, Hefei, China; ^3^Department of Clinical Medicine, The Second School of Clinical Medical, Anhui Medical University, Hefei, China; ^4^Teaching Center for Preventive Medicine, School of Public Health, Anhui Medical University, Hefei, China; ^5^Department of Electrocardiogram, The Second Hospital of Anhui Medical University, Hefei, China; ^6^Department of Orthopedics Trauma, The First Affiliated Hospital of Anhui Medical University, Hefei, China

**Keywords:** osteoarthritis, hormonal reproductive factors, Mendelian randomization, causality, GWAS

## Abstract

**Objectives:**

Hormonal reproductive factors have been considered to play an important role in the etiology of osteoarthritis (OA). We performed Mendelian randomization (MR) to examine whether a causal effect existed between them.

**Methods:**

MR was performed by using publicly released genome-wide association study (GWAS) summary statistics to estimate the causal associations of three relevant exposures, including age at menarche (AAM), age at natural menopause (ANM) and age at first birth (AFB), with the risk of OA. We employed several MR methods, including inverse-variance weighted (IVW), MR-Egger regression, weighted median and weighted mode, to estimate the causality. We performed a sensitivity analysis by manually pruning pleiotropic variants associated with the known confounder body mass index (BMI).

**Results:**

The instrumental variables that achieved genome-wide significance, including 349 AAM single nucleotide polymorphisms (SNPs), 121 AAM SNPs, 54 ANM SNPs, and 10 AFB SNPs, were incorporated into the operation. IVW analysis indicated that each additional year in AFB was associated with a decreasing risk of hip and/or knee OA and overall OA (hip and/or knee OA: OR = 0.79, 95% CI: 0.64–0.93, *P* = 1.33 × 10^−3^; overall OA: OR = 0.80, 95% CI: 0.68–0.92, *P* = 1.80 × 10^−4^). In addition, our results suggested that AAM exerted a causal effect on knee OA in an unfavorable manner (OR = 0.86, 95% CI: 0.76–0.95, *P* = 1.58 × 10^−3^). After accounting for the effect of BMI, the causal effect association between AFB and hip and/or knee OA was also examined (IVW: OR = 0.78, 95% CI: 0.66–0.92, *P* = 3.22 × 10^−3^).

**Conclusion:**

Our findings add a growing body of evidence surrounding the unfavorable effects of early AFB on OA risk, suggesting the essential for relevant health problem management in susceptible populations.

## Introduction

Osteoarthritis (OA) is the most common form of joint disease around the world, and it is estimated that approximately 302 million individuals suffer from OA (1). Numerous studies have shown that OA has been ranked as the leading cause of disability and accounts for a heavy burden of disease (2, 3). As the aging population and obesity pandemic increase, OA is more prevalent and has become a major public health concern (4). However, the exact mechanism underlying the pathogenesis of OA has not been fully clarified.

Epidemiological studies indicate a distinct sexual disparity in the incidence of OA in which females develop the disease more frequently and more severely than males, particularly after menopausal age (5). Individuals who had undergone postmenopausal hormone therapy exhibited a higher risk of developing OA than those who did not, revealing that hormonal reproductive factors may play a critical role in the initiation and progression of OA. There is a wealth of data concerning the associations of hormonal reproductive factors, including age at menarche (AAM), age at nature menopause (ANM) and age at first birth (AFB), with incident OA. For example, findings from a prospective cohort study suggested that an early AAM increased the risk of hip and knee replacement for OA, while the ANM was not associated with the risk (6). Another cross-sectional study demonstrated that women with ANM < 45 years were associated with a 2.60-fold risk of developing OA compared with those with an ANM ≥ 45 years (7). These discrepancies may be due to potential confounding factors (such as obesity and age) and reverse causality. Reproductive behavior is shaped by biology and environment, while AFB represents an accurate measure of complex reproductive outcomes, are frequently recorded and consistently measured. It is indicated that the heritability of AFB shifted from 9% for women born in 1940 to 22% in 1965 (8).

Mendelian randomization (MR) is an epidemiological method that utilizes genetic variants robustly associated with exposure as instrumental variables (IVs) to estimate the causal effect of exposure on an outcome (9, 10). Because of the bias from confounders, reverse causation and measurement error, even there was a significant statistical association between the exposure and outcome, traditional analyses have limitations for the assessment of causality, while MR offers an alternative way to probe it. It is considered important to target the management of relevant health problems in OA susceptible individuals, which requires us to first clarify the root causes of OA. In this study, confounders including environmental factors and BMI could be excluded due to the application of MR. To the best of our knowledge, the MR study focusing on this topic do not exist. Therefore, it is necessary to do the MR design to investigate it.

## Materials and methods

### Study design and data source

In our current study, a standard two-sample framework was applied to explore the effect of three female hormonal reproductive factors (AAM, ANM and AFB) on hospital-diagnosed OA and its subtypes (OA at any site, hip OA, knee OA, hip and/or knee OA). Individuals were restricted to European ancestry to decrease the bias from population stratification. As a milestone in the development of female pubertal development, age at menarche varies markedly among females. GWAS have identified tens of thousands of sequence variant on a genome-wide scale in humans and from which to determine the effect size of genetic variants statistically in order to identify the risk factors of disease etiology in different ethnic populations. GWAS gives us the opportunity to research complex diseases by comparing SNPs loci detected genome-wide in patients to controls for all variant allele frequencies, obviating the need to presuppose causative genes as in a candidate gene strategy. Genetic associations with AAM were obtained from two large GWAS meta-analyses, including a total of 329,345 individuals in AAM (11) and 182,416 in AAM (12). Summary level statistics for ANM were derived from the GWAS of 69,360 women, identifying 44 genomic regions containing 54 independent signals, most of which were associated with one or more DNA damage response pathway genes (13). Biological processes, such as AFB, are indicated to partly cause reproductive behavior. A recent GWAS of 251,151 women examined the genetic architecture of reproductive rhythms defined by AFB, and 10 AFB-associated loci were identified (14). The full OA (OA at any site, hip OA, knee OA, hip and/or knee OA) summary statistics were obtained from the largest release GWAS meta-analysis across 16.5 million derived from the UKB resource (15). Accounting for the confounding effects of other traits that were genetically correlated with sleep phenotypes, sensitivity analysis was performed after adjusting for BMI-related genetic disorders. Summary statistics of BMI were downloaded from a GWAS including 806,834 individuals (16). A more detailed description of the included data sources is available in Supplementary Table 1. No ethical approval was required in this work, as all the data analyzed were publicly available.

### Selection of the genetic instruments

A valid IV estimator should meet the following three assumptions: (1) reliably and strongly associate with the risk factor for interest (relevance assumption); (2) no unmeasured confounders of the associations between genetic variants and outcome (independence assumption); and (3) independent of the outcome (exclusion restriction) (17). The qualified IVs were selected as follows: After removing SNPs with missing information, a list of SNPs passing the threshold of significance *P* < 5 × 10^−8^ was first screened using a distance-based metric. We performed PLINK to calculate *r*^2^ between all selected SNPs in European ancestry samples from the 1000 Genomes Project (18). To ensure that all the selected SNPs obeyed the independence assumption, only those with the smallest *P* value were retained among all pairs of SNPs with *r*^2^ > 0.01. A proxy SNP in strong LD (*r*^2^ > 0.8) was included where a specific instrument SNP was not available in the look-up GWAS dataset. To ensure that all corresponding risk factors and outcome alleles were on the same strand, we harmonized the effect of these instrumental SNPs where possible. The equation R^2^ = 2^*^Beta^∧^2^*^EAF^*^(1-EAF)/(2^*^Beta^∧^2^*^EAF^*^(1-EAF)+2^*^SE^∧^2^*^SampleSize^*^EAF^*^(1-EAF), F = R^2^(SampleSize-2)/(1 – R^2^) was used to calculate the F-statistic for all selected instrument SNPs separately and synthetically to reject the weak instruments with an F-statistic lower than 10 (19). R^2^ in the equation represents the individual exposure variance explained by each IV.

### Mendelian randomization analysis

Subsequently, MR analyses were conducted with inverse variance weighted (IVW), MR Egger regression, weighted median and weighted mode. The primary calculation was run by inverse variance weighted, which estimates the ratio from several instruments. This method assumed that all SNPs were valid instruments or were invalid with zero overall bias. However, IVW may be overpraised in the presence of heterogeneity that can occur due to, among other factors, horizontal pleiotropy or, more simply, off-target genetic effects.

Consistency in results across methods builds confidence in the obtained estimates, as they depend on different assumptions and models of horizontal pleiotropy. MR-Egger deemed uncorrelated associations between SNP exposure and horizontal pleiotropic effects, which indicates instrument strength independent of the direct effects assumption. MR-Egger regression analysis, whose slope represents the causal effect estimate, is robust to invalid instruments against directional pleiotropy (20, 21). A weighted median requires the weight of each SNP in the overall estimate to depend on the precision of its ratio estimate, which differs from a simple median estimate. More specifically, 50% of the weights come from valid IVs smaller than or equal to the weighted median in this analysis (22), while the weighted mode requires that the largest subset of instruments which identify the same causal effect to be valid instruments (23).

### Pleiotropy and sensitivity analysis

Although MR is a potentially powerful technique for strengthening causal inference, several issues, including disequilibrium, pleiotropy and epigenetic effects, could disturb instrumental variable assumptions. Funnel plots were used as a visual test for horizontal pleiotropy, where symmetry is indicative of a lower probability of pleiotropy (24). As an additional control for pleiotropy, we applied the global test, outlier test, and distortion test using the MR pleiotropy residual sum and outlier (MR-PRESSO) to identify and correct for outliers in IVW linear regression (25). Furthermore, MR-Egger regression provides an estimate of the average pleiotropy effect, and an intercept of the regression equation of 0 proves the evidence of pleiotropy (26). In the regression model, regression coefficients are highly susceptible to an individual datapoint. Leave-one-out sensitivity analysis was performed to identify whether the association was disproportionately influenced by a single SNP. An increased BMI is a well-known risk factor for OA (15). To minimize the possibility of spurious causal associations due to confounding factor BMI, we performed a sensitivity analysis by manually pruning pleiotropic BMI-associated instrumental variables.

### Statistical analysis

We employed the packages “Two Sample MR” (24) and “Mendelian Randomization” (27) to perform MR analysis. Forest plots were produced using the “forestplot” package. The Bonferroni method was utilized in the primary analysis to indicate multiple comparisons. Correcting for 3 exposures and 4 outcomes, *P* value below 0.004 indicated strong evidence of associations (0.05/12 = 0.004). All statistical analyses were implemented in R project version 3.6.1.

## Results

In total, after implementing the pruning strategy previously described, there were 349 SNPs achieved genome-wide significance for AAM (11) and 121, 54 and 10 IVs for AAM (12), ANM and AFB, respectively. F-statistic values for individual instrumental SNPs were all above the threshold 10, with means of 64.27, 58.00, 68.14 and 36.49 for AAM (11), AAM (12), ANM and AFB, respectively (Table 1). SNPs were excluded or substituted with highly correlated (*r*^2^ > 0.8) proxy SNPs due to unavailability in outcome datasets or palindromic with ambiguous A/T or G/C. Detailed information for incorporated instrumental SNPs is presented in Supplementary Tables 2–4.

**Table 1:** Univariable MR results of hormonal reproductive factors on risk of OA and subtypes.

**Exposure**	**Outcome**	**No. of SNPs**	**F-Statistic**	**OR (95% CI)**	***P***
AAM (11)	Overall OA	336	64.27	0.91 (0.85–0.98)	5.95E-03
	Hip OA	337		1.01 (0.89–1.13)	8.56E-01
	Knee OA	337		0.86 (0.76–0.95)	1.58E-03
	Hip and/or knee OA	337		0.92 (0.84–1.00)	3.64E-02
AAM (12)	Overall OA	119	58.00	0.94 (0.86–1.03)	2.09E-01
	Hip OA	119		1.16 (0.97–1.34)	1.23E-01
	Knee OA	119		1.02 (0.85–1.19)	2.31E-01
	Hip and/or knee OA	119		1.02 (0.85–1.19)	6.99E-01
ANM	Overall OA	51	68.14	1.00 (0.97–1.03)	9.05E-01
	Hip OA	51		0.98 (0.92–1.03)	4.43E-01
	Knee OA	51		1.00 (0.96–1.04)	9.46E-01
	Hip and/or knee OA	51		1.00 (0.97–1.04)	7.95E-01
AFB	Overall OA	10	36.49	0.80 (0.68–0.92)	1.80E-04
	Hip OA	10		0.76 (0.51–1.00)	2.65E-02
	Knee OA	10		0.81 (0.63–0.98)	1.57E-02
	Hip and/or knee OA	10		0.79 (0.64–0.93)	1.33E-03
AAM (11) no BMI	Overall OA	285	62.99	0.96 (0.90–1.03)	3.01E-01
	Hip OA	286		1.09 (0.96–1.24)	1.86E-01
	Knee OA	286		1.01 (0.88–1.16)	8.90E-01
	Hip and/or knee OA	286		0.96 (0.86–1.06)	4.15E-01
AAM (12) no BMI	Overall OA	90	58.44	1.01 (0.92–1.10)	9.04E-01
	Hip OA	90		1.23 (1.01–1.49)	4.28E-02
	Knee OA	90		1.06 (0.92–1.22)	4.10E-01
	Hip and/or knee OA	90		1.09 (0.97–1.22)	1.60E-01
AFB no BMI	Overall OA	8	34.02	0.83 (0.72–0.94)	4.91E-03
	Hip OA	8		0.77 (0.59–1.02)	6.85E-02
	Knee OA	8		0.80 (0.65–0.98)	2.83E-02
	Hip and/or knee OA	8		0.78 (0.66–0.92)	3.22E-03

OA: osteoarthritis; AAM: age at menarche; ANM: age at natural menopause; AFB: age at first birth; BMI: body mass index; SNPs: single nucleotide polymorphisms.

**Figure 1:**
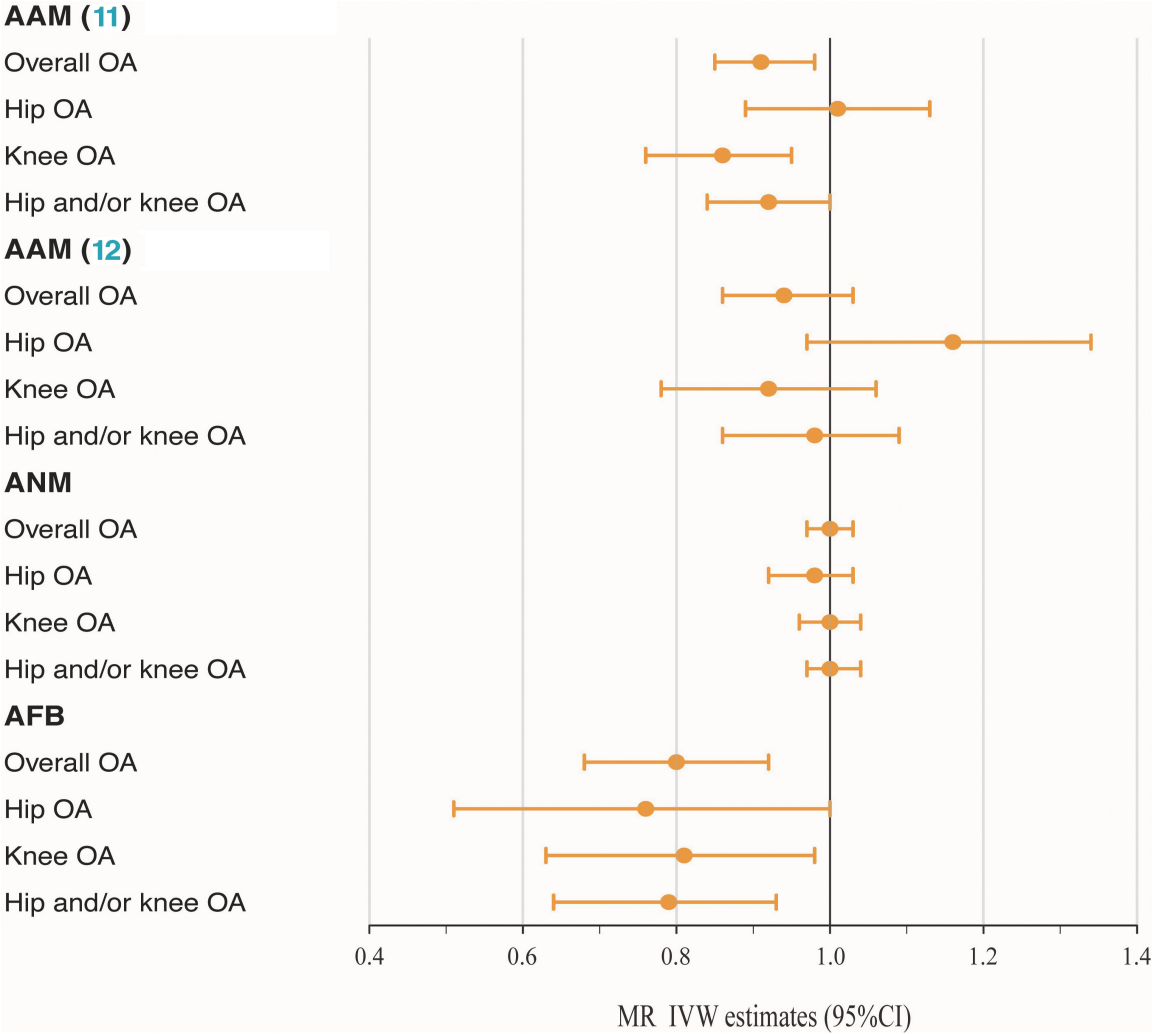
Forest plot of hormonal reproductive factors on the risk of OA and subtypes in IVW estimates.

The MR analysis indicated that genetically determined each additional year in AAM (11) was associated with a decreasing risk of knee OA after correcting for multiple testing (IVW: OR = 0.86, 95% CI: 0.76–0.95, *P* = 1.58 × 10^−3^). The causality between AAM (11) and overall OA (*P* = 5.95 × 10^−3^) and hip and/or knee OA (*P* = 3.64 × 10^−2^) was only normally significantly positive, with IVW OR_per − SDincrement(95%CI)_ of 0.91 (0.85–0.98) and 0.92 (0.84–1.00), respectively. To the best of our knowledge, such causality was not observed in another AAM (12) exposures, with *P* values above the threshold and OR of 0.94, 1.16, 1.02 and 1.02 for OA and three subtypes, respectively. The available evidence also made it difficult to explain the causality of ANM to OA and subtypes. Furthermore, the IVW method indicated that AFB exerted a causal effect on OA and all subtypes in an unfavorable manner, with OR of 0.80 and 0.79 for overall (95% CI: 0.68–0.92, *P* = 1.80 × 10^−4^) and hip and/or knee OA (95% CI: 0.64–0.93, *P* = 1.33 × 10^−3^), respectively. However, as shown in Table 1 and Figure 1, some nominally significant positive causality correlations with OA did not pass multiple-testing correction for the Bonferroni method (hip OA: OR = 0.76, 95% CI: 0.51–1.00, *P* = 2.65 × 10^−2^; knee OA: OR = 0.81, 95% CI: 0.63–0.98, *P* = 1.57 × 10^−2^). Additional methods, including weighted median, weighted mode and MR-Egger section, validate the uniformity conclusion (Supplementary Table 5).

To effectively control pleiotropy, we next investigated the MR-PRESSO and *p* value for the MR-Egger intercept test. The sensitivity analysis revealed the absence of outliers (Supplementary Table 7) and horizontal pleiotropy (Supplementary Figure 2). As shown in Supplementary Figure 2, symmetry in funnel plots did not show any evidence of publication bias. In addition, the leave-one-out analysis showed that none of the individual genetic markers drove the majority of the association signal. The scatter plots for effect sizes of SNPs for three hormonal related exposures and those for OA and subtypes are shown in Supplementary Figure 1.

**Figure 2:**
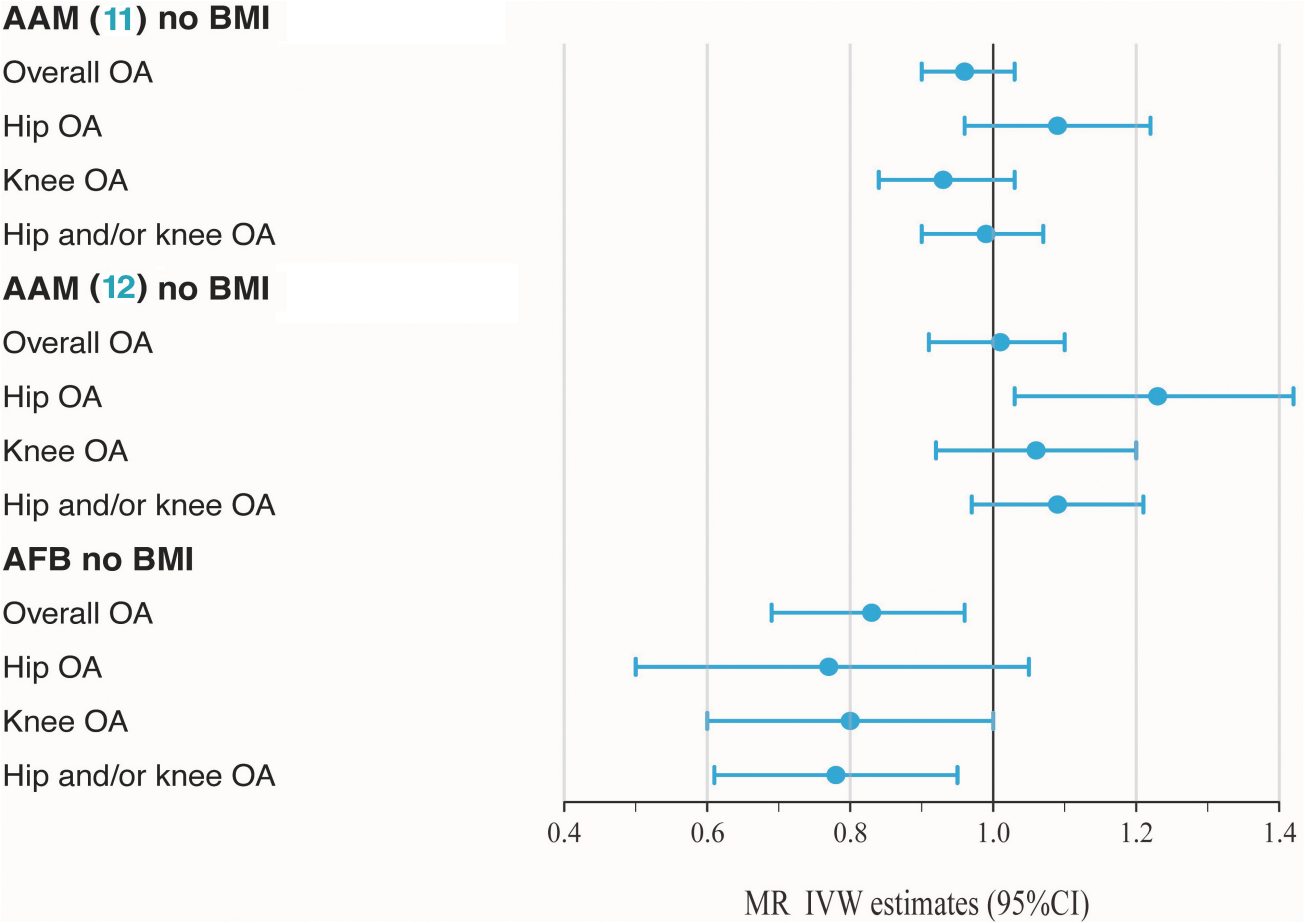
Forest plot of hormonal reproductive factors on risk of OA and subtypes in IVW estimates adjusting for BMI.

BMI is a known modifiable risk factor that plays an important role in the etiology of OA and shapes reproductive exposures. A total of 82 SNPs were found to be associated with BMI (*P* < 5 × 10^−8^), including 51 in AAM (11), 29 in AAM (12) and 2 in AFB. SNPs associated with BMI have been annotated with ^*^ in the Supplementary Tables 2–4. As shown in Table 1 and Figure 2, no casualty between AAM (11) no BMI and keen OA were found when we performed MR again (IVW: OR = 1.01, 95% CI: 0.88–1.16, *P* = 0.89), which indicates that BMI-associated SNPs confounded causality in our initial calculation. The negative result was confirmed in AAM (12) no BMI. Similarly, after deleting two BMI-associated SNPs, there was not enough evidence to indicate the causal relationship between AFB no BMI and overall OA (IVW: OR = 0.83, 95% CI: 0.72–0.94, *P* = 4.91 × 10^−3^). However, a similar causal effect association between AFB and hip and/or knee OA could still be measured (IVW: OR = 0.78, 95% CI: 0.66–0.92, *P* = 3.22 × 10^−3^). Additional methods section validate the uniformity conclusion (Supplementary Table 6).

## Discussion

In the current study, we performed two-sample MR to investigate the causality between three hormonal reproductive factors and the risk of OA. The concern was that 1 year later in AFB was associated with a reduced risk of OA. These findings support the hypothesis that AFB may play a causal role in the pathway of developing OA. The adverse causal effects were robust in our MR after pruning potential confounder BMI.

Several observational studies confirm the causal association of hormonal reproductive factors with OA, while current results on this topic from conventional epidemiological studies remain controversial. A large cohort study with 30.727 cases is consistent with our conclusions, affirming the role of AFB in OA etiology (15). Other studies also point toward some positive association, and a prospective cohort study containing over 30,000 women found that older age at menarche was associated with a decreased risk of total knee replacement (TKR) due to primary OA (28). Researchers proposed that one possible explanation could be that lower AAM may be a marker of other factors, such as higher BMI when young (6). High BMI is known as a risk factor for OA, but it is unlikely that BMI would explain the casualty found here, as we adjusted our analyses for BMI, and our findings were consistently observed within subgroups of no BMI. However, the assumed relationship between the female hormonal aspects and OA was not clinically significant in another cohort study (29). Since hormonal reproductive factors are prone to bias due to interference from potential confounders which difficult to be excluded by traditional epidemiology, MR estimates reflect the causality at the genetic level.

Given the complexity of these confounders, the underlying mechanism of hormonal reproductive factors in the development of OA remains to be elucidated. Estrogen is considered to strongly associate with the female hormonal reproductive cycle, in which receptors are found on bone and chondrocyte cells (30). Several studies show evidence of the associations between radiographic changes in OA and high bone density since considering estrogen could prevent bone loss (31, 32). Consequently, greater exposure to estrogen, while preventing bone loss, may plausibly promote OA. Gao et al. studied estrogen and estrogen metabolites in Chinese women with OA. Compared to the controls (healthy and rheumatoid arthritis women), premenopausal women with OA had a significantly lower concentration of 2-hydroxyestrone and free estrogen in serum. In postmenopausal women, the serum concentration of 2-hydroxyestradiol was increased compared to that in controls, while free and total estrogen were significantly decreased. Apart from estrogen deficiency, rapidly elevated serum levels of 2-hydroxyestrone in the perimenopausal period may correlate with the pathogenesis of OA (33, 34). Furthermore, it has been reported that estrogen may have different effects on the initiation and progression of OA. Hence, it is difficult to ratiocinate the biological mechanisms that underlie this study due to these heterogeneous effects. However, the effects of female hormones on OA can be further explored in animal models and *in vitro* studies.

Our study has several strengths. The large sample size and richness of the data set for reproductive variables of interest led the estimated effects to be close to the truth. In addition, three different reproductive traits (AAM, ANM, and AFB) were incorporated to reflect the length of the reproductive period and complementing each other well. To reduce the interference of potential factors, we examined OA directly rather than proxies of OA, such as hospitalization or joint replacement. Moreover, we were able to adjust for not only hormonal reproductive factors but also reported confounders of OA, such as BMI. Notwithstanding, we must acknowledge several limitations. Firstly, there was no stratification of sex in the existing GWAS data set, while our selection of hormonal reproductive factors was female specific. However, since most cases in the GWAS dataset were from females (63.7% in overall OA), we thought the estimated effects would be close to the truth. Secondly, to diminish population stratification, our samples were restricted to the European population, which leads our findings to be applicable for European populations. Finally, the design of our study precluded us from considering other factors, such as environmental effects and hormone use, in addition to the only confounder BMI regarded in the current study.

In summary, our findings add to a growing body of evidence surrounding the unfavorable effects of early age at first birth on OA risk, suggesting the essential for relevant health problem management in susceptible populations. Further large-scale studies or longitudinal studies are required to validate our findings.

## Data availability statement

The original contributions presented in the study are included in the article/Supplementary material, further inquiries can be directed to the corresponding author.

## Ethics statement

Each participating study obtained written informed consent from all participants and received approval from the appropriate local institutional review boards.

## Author contributions

ZY was responsible for the conception, design of the study, full access to all the data in the study, took responsibility for the integrity of the data, and the accuracy of the data analysis. BW and JW Performed the statistical analysis and drafted the manuscript. All authors were involved in drafting the article or revising it critically for important intellectual content, and approved the final version to be published.

## Funding

This study was supported by the University Natural Foundation of Anhui Educational Committee (KJ2020A0199) and Nature Science Foundation of Anhui Medical University (2021xkj156).

## Conflict of interest

The authors declare that the research was conducted in the absence of any commercial or financial relationships that could be construed as a potential conflict of interest.

## Publisher's note

All claims expressed in this article are solely those of the authors and do not necessarily represent those of their affiliated organizations, or those of the publisher, the editors and the reviewers. Any product that may be evaluated in this article, or claim that may be made by its manufacturer, is not guaranteed or endorsed by the publisher.

## Abbreviations

OA: osteoarthritis
MR: Mendelian randomization
GWAS: genome-wide association studies
AAM: age at menarche
ANM: age at natural menopause
AFB: age at first birth
IVW: inverse-variance weighted
BMI: body mass index
SNPs: single nucleotide polymorphisms
MR-PRESSO: MR pleiotropy residual sum and outlier
TKR: total knee replacement.
