# Supplementary material for: Using genetic instruments to estimate the causal effect of hormonal reproductive factors on osteoarthritis
Source: Front Public Health. 2022 Nov 14;10:941067. doi: 10.3389/fpubh.2022.941067 (PMC9702564; doi:10.3389/fpubh.2022.941067)
Supplement: Supplementary file 1 [file Data_Sheet_1.docx]

**Supplementary Table 1:** Summary of genome-wide association studies (GWAS) datasets in our study.

| **Phenotype** | **Type of trait** | **Author, published year** | **Consortium** | **Sample size** | **No. of cases (Binary trait)** | **proportion of female (%)** | **PMID** |
| --- | --- | --- | --- | --- | --- | --- | --- |
| Overall OA | Binary | Zengini E, 2018 | UKB | 50508 | 10083 | 63.7 | 29559693 |
| Hip OA | Binary | Zengini E, 2018 | UKB | 11989 | 2396 | 54.3 | 29559693 |
| Knee OA | Binary | Zengini E, 2018 | UKB | 22347 | 4462 | 47.6 | 29559693 |
| Hip and/or knee OA | Binary | Zengini E, 2018 | UKB | 32970 | 6586 | 49.9 | 29559693 |
| AAM (11) | Continuous | Felix R Day et al, 2017 | ReproGen, 23andMe, UKB | 329,345 | NA | NA | 28436984 |
| AAM (12) | Continuous | John Rb Perry et al, 2014 | ReproGen, BCAC, GIANT, GEOFS, EGG | 182,416 | NA | NA | 25231870 |
| ANM | Continuous | Felix R Day et al, 2015 | BCAC, ICOGs, PRACTICAL | 69,360 | NA | NA | 26414677 |
| AFB | Continuous | Nicola Barban et al, 2016 | GIANT | 251,151 | NA | NA | 27798627 |
| BMI | Continuous | Pulit SL et al, 2019 | UKB, GIANT, DIAGRAM, GERA | 806,834 | NA | NA | 30239722 |

OA: osteoarthritis; AAM: age at menarche; ANM: age at natural menopause; AFB: age at first birth; BMI: body mass index

**Supplementary Table 2:** Detailed information for instrumental variables of age at menarche (AAM).

| **Chr** | **Position**  **(hg19)** | **rsID** | **Effect Allele** | **Other Allele** | **Exposure** | | | | **Outcome** | | | | **R^2^** | **F-statistics** |
| --- | --- | --- | --- | --- | --- | --- | --- | --- | --- | --- | --- | --- | --- | --- |
|  |  |  |  |  | **EAF** | **Beta** | **SE** | **p** | **EAF** | **Beta** | **SE** | **p** |  |  |
| **AAM (11)** | | | | | | | | | | | | | | |
| 6 | 105397418 | rs395962 | T | G | 0.316 | 0.127 | 0.004 | 1.00E-200 | 0.318 | -0.010 | 0.017 | 5.49E-01 | 3.05E-03 | 1008 |
| 9 | 108941509 | rs10156597 | A | T | 0.677 | 0.102 | 0.004 | 5.04E-139 | 0.686 | 0.022 | 0.017 | 1.93E-01 | 1.97E-03 | 650 |
| 11 | 122813983 | rs7114175 | A | T | 0.496 | -0.060 | 0.004 | 1.14E-56 | 0.501 | 0.016 | 0.016 | 3.12E-01 | 6.83E-04 | 225 |
| 2 | 625029 | rs7576624* | T | C | 0.826 | -0.074 | 0.005 | 4.98E-50 | 0.828 | 0.062 | 0.021 | 3.52E-03 | 6.65E-04 | 219 |
| 1 | 165398744 | rs157877 | A | G | 0.127 | -0.084 | 0.006 | 2.25E-48 | 0.126 | 0.013 | 0.024 | 5.75E-01 | 5.95E-04 | 196 |
| 3 | 117552111 | rs10934420 | T | C | 0.505 | -0.055 | 0.004 | 1.54E-47 | 0.507 | -0.017 | 0.016 | 2.90E-01 | 5.74E-04 | 189 |
| 1 | 177894287 | rs506589* | T | C | 0.803 | 0.070 | 0.005 | 1.54E-47 | 0.791 | -0.041 | 0.019 | 3.49E-02 | 5.95E-04 | 196 |
| 9 | 114318394 | rs7852169 | C | G | 0.912 | -0.097 | 0.007 | 1.82E-46 | 0.918 | 0.002 | 0.029 | 9.32E-01 | 5.83E-04 | 192 |
| 17 | 49613785 | rs9635759 | A | G | 0.307 | 0.059 | 0.004 | 2.78E-46 | 0.304 | -0.012 | 0.017 | 4.72E-01 | 6.60E-04 | 218 |
| 18 | 44748467 | rs1512238 | A | G | 0.421 | -0.054 | 0.004 | 2.48E-44 | 0.421 | -0.009 | 0.016 | 5.86E-01 | 5.53E-04 | 182 |
| 16 | 69733460 | rs7359336* | A | G | 0.579 | -0.053 | 0.004 | 5.33E-44 | 0.588 | 0.016 | 0.016 | 3.24E-01 | 5.33E-04 | 176 |
| 14 | 60943106 | rs10138913 | T | C | 0.306 | 0.056 | 0.004 | 1.14E-41 | 0.301 | 0.018 | 0.017 | 2.88E-01 | 5.95E-04 | 196 |
| 2 | 156621725 | rs142058842 | C | G | 0.830 | -0.068 | 0.005 | 2.42E-40 | 0.837 | -0.031 | 0.021 | 1.41E-01 | 5.61E-04 | 185 |
| 2 | 56588406 | rs111567162 | A | T | 0.174 | 0.068 | 0.005 | 2.49E-40 | 0.164 | 0.039 | 0.021 | 6.65E-02 | 5.61E-04 | 185 |
| 16 | 53814363 | rs9972653* | T | G | 0.400 | -0.051 | 0.004 | 6.47E-40 | 0.398 | 0.036 | 0.016 | 2.79E-02 | 4.93E-04 | 162 |
| 2 | 199621641 | rs10931831 | T | C | 0.356 | -0.053 | 0.004 | 1.87E-39 | 0.343 | 0.003 | 0.017 | 8.37E-01 | 5.33E-04 | 176 |
| 1 | 74977870 | rs1040070* | C | G | 0.565 | 0.050 | 0.004 | 7.01E-38 | 0.567 | -0.003 | 0.016 | 8.66E-01 | 4.74E-04 | 156 |
| 7 | 41470093 | rs1079866 | C | G | 0.863 | -0.071 | 0.006 | 3.65E-37 | 0.867 | -0.039 | 0.023 | 8.89E-02 | 4.25E-04 | 140 |
| 2 | 199756278 | rs13023912 | A | G | 0.652 | -0.051 | 0.004 | 4.82E-36 | 0.659 | 0.019 | 0.017 | 2.50E-01 | 4.93E-04 | 162 |
| 16 | 14388750 | rs1704528 | T | C | 0.662 | -0.051 | 0.004 | 1.50E-35 | 0.669 | -0.049 | 0.017 | 3.62E-03 | 4.93E-04 | 162 |
| 14 | 100846991 | rs12894936 | T | C | 0.294 | -0.052 | 0.004 | 3.73E-34 | 0.267 | 0.030 | 0.018 | 9.55E-02 | 5.13E-04 | 169 |
| 8 | 4560227 | rs2724961 | T | C | 0.468 | -0.046 | 0.004 | 3.76E-33 | 0.470 | 0.024 | 0.016 | 1.28E-01 | 4.01E-04 | 132 |
| 2 | 105870779 | rs2679894 | A | G | 0.437 | 0.051 | 0.004 | 5.37E-33 | 0.425 | -0.025 | 0.016 | 1.22E-01 | 4.93E-04 | 162 |
| 18 | 3813464 | rs11873906 | A | G | 0.720 | -0.051 | 0.004 | 2.33E-32 | 0.723 | -0.002 | 0.018 | 8.99E-01 | 4.93E-04 | 162 |
| 2 | 73535526 | rs34437050 | A | G | 0.012 | 0.241 | 0.020 | 4.04E-32 | 0.015 | 0.048 | 0.065 | 4.61E-01 | 4.41E-04 | 145 |
| 19 | 9984509 | rs4804117 | T | G | 0.557 | 0.046 | 0.004 | 3.58E-31 | 0.549 | -0.012 | 0.016 | 4.47E-01 | 4.01E-04 | 132 |
| 3 | 86910329 | rs9758500 | A | G | 0.375 | -0.046 | 0.004 | 1.36E-30 | 0.379 | 0.028 | 0.016 | 8.22E-02 | 4.01E-04 | 132 |
| 9 | 86715566 | rs7853970 | T | C | 0.464 | 0.045 | 0.004 | 2.19E-30 | 0.463 | -0.021 | 0.016 | 1.88E-01 | 3.84E-04 | 127 |
| 2 | 466003 | rs62104180* | A | G | 0.051 | 0.113 | 0.010 | 3.98E-29 | 0.050 | -0.086 | 0.037 | 1.97E-02 | 3.88E-04 | 128 |
| 11 | 13324530 | rs10832021* | A | G | 0.710 | -0.047 | 0.004 | 6.27E-29 | 0.713 | -0.010 | 0.017 | 5.69E-01 | 4.19E-04 | 138 |
| 3 | 185651469 | rs2300922 | T | C | 0.414 | 0.043 | 0.004 | 1.11E-28 | 0.429 | -0.043 | 0.016 | 7.60E-03 | 3.51E-04 | 116 |
| 9 | 109148074 | rs56927240 | T | C | 0.201 | -0.057 | 0.005 | 8.50E-28 | 0.057 | -0.063 | 0.043 | 1.38E-01 | 3.94E-04 | 130 |
| 11 | 101438191 | rs6590889 | T | C | 0.340 | -0.044 | 0.004 | 8.62E-28 | 0.331 | -0.011 | 0.017 | 5.10E-01 | 3.67E-04 | 121 |
| 6 | 126727908 | rs4897178 | T | G | 0.555 | 0.043 | 0.004 | 1.02E-27 | 0.544 | 0.015 | 0.016 | 3.57E-01 | 3.51E-04 | 116 |
| 3 | 24715135 | rs1984870 | T | G | 0.473 | 0.042 | 0.004 | 5.61E-27 | 0.468 | 0.012 | 0.016 | 4.51E-01 | 3.35E-04 | 110 |
| 12 | 50263148 | rs7132908* | A | G | 0.388 | -0.042 | 0.004 | 6.64E-27 | 0.387 | 0.028 | 0.016 | 8.32E-02 | 3.35E-04 | 110 |
| 15 | 23794517 | rs7178532 | A | G | 0.685 | 0.044 | 0.004 | 2.29E-26 | 0.692 | 0.016 | 0.017 | 3.45E-01 | 3.67E-04 | 121 |
| 8 | 78116203 | rs10094506 | T | C | 0.281 | -0.045 | 0.004 | 2.46E-26 | 0.283 | 0.027 | 0.017 | 1.28E-01 | 3.84E-04 | 127 |
| 6 | 100158873 | rs6931884 | T | C | 0.129 | 0.059 | 0.006 | 1.64E-25 | 0.133 | -0.002 | 0.023 | 9.30E-01 | 2.94E-04 | 97 |
| 13 | 112186283 | rs9522262* | C | G | 0.492 | 0.041 | 0.004 | 1.69E-25 | 0.489 | -0.014 | 0.016 | 3.68E-01 | 3.19E-04 | 105 |
| 10 | 1730008 | rs7907759 | A | G | 0.471 | 0.041 | 0.004 | 2.43E-25 | 0.482 | 0.004 | 0.016 | 7.86E-01 | 3.19E-04 | 105 |
| 15 | 89042467 | rs12915845 | T | C | 0.424 | -0.040 | 0.004 | 3.70E-25 | 0.424 | 0.024 | 0.016 | 1.31E-01 | 3.04E-04 | 100 |
| 6 | 41893323 | rs9349203 | A | G | 0.546 | -0.040 | 0.004 | 5.93E-25 | 0.550 | 0.005 | 0.016 | 7.51E-01 | 3.04E-04 | 100 |
| 15 | 67987293 | rs10153031 | T | G | 0.406 | 0.040 | 0.004 | 6.05E-25 | 0.397 | -0.013 | 0.016 | 4.12E-01 | 3.04E-04 | 100 |
| 15 | 60781513 | rs3743266 | T | C | 0.668 | 0.042 | 0.004 | 8.52E-25 | 0.668 | 0.004 | 0.017 | 8.29E-01 | 3.35E-04 | 110 |
| 4 | 104640935 | rs3733632 | A | G | 0.844 | -0.054 | 0.005 | 1.04E-24 | 0.848 | 0.027 | 0.022 | 2.30E-01 | 3.54E-04 | 117 |
| 3 | 49568181 | rs115435316 | A | G | 0.033 | 0.115 | 0.011 | 1.67E-24 | 0.035 | 0.062 | 0.042 | 1.43E-01 | 3.32E-04 | 109 |
| 1 | 72750500 | rs11209943* | A | G | 0.391 | 0.038 | 0.004 | 1.08E-22 | 0.397 | -0.015 | 0.016 | 3.42E-01 | 2.74E-04 | 90 |
| 19 | 47609223 | rs4804025* | A | G | 0.704 | -0.041 | 0.004 | 3.11E-22 | 0.715 | 0.016 | 0.018 | 3.55E-01 | 3.19E-04 | 105 |
| 11 | 8404501 | rs16937956* | A | G | 0.638 | -0.038 | 0.004 | 6.38E-22 | 0.652 | 0.016 | 0.017 | 3.33E-01 | 2.74E-04 | 90 |
| 16 | 29896390 | rs8051833* | A | G | 0.342 | -0.040 | 0.004 | 9.26E-22 | 0.348 | 0.015 | 0.017 | 3.72E-01 | 3.04E-04 | 100 |
| 4 | 95143122 | rs3113862 | A | G | 0.599 | -0.037 | 0.004 | 9.69E-22 | 0.596 | 0.023 | 0.016 | 1.56E-01 | 2.60E-04 | 86 |
| 6 | 100194846 | rs9403051 | A | G | 0.570 | 0.037 | 0.004 | 1.76E-21 | 0.573 | 0.002 | 0.016 | 9.01E-01 | 2.60E-04 | 86 |
| 1 | 44029353 | rs11210871 | C | G | 0.298 | 0.040 | 0.004 | 3.07E-21 | 0.300 | 0.008 | 0.017 | 6.50E-01 | 3.04E-04 | 100 |
| 20 | 17109159 | rs852061 | A | C | 0.365 | -0.037 | 0.004 | 1.58E-20 | 0.359 | 0.023 | 0.016 | 1.54E-01 | 2.60E-04 | 86 |
| 22 | 49678782 | rs8136272 | A | T | 0.728 | 0.040 | 0.004 | 6.18E-20 | 0.730 | 0.006 | 0.018 | 7.54E-01 | 3.04E-04 | 100 |
| 6 | 77713859 | rs1414186 | T | G | 0.205 | -0.043 | 0.005 | 1.99E-19 | 0.204 | 0.035 | 0.019 | 7.06E-02 | 2.25E-04 | 74 |
| 9 | 76905178 | rs2604265 | A | G | 0.266 | 0.039 | 0.004 | 2.50E-19 | 0.266 | -0.015 | 0.018 | 4.14E-01 | 2.89E-04 | 95 |
| 6 | 54640512 | rs9474996 | A | T | 0.555 | -0.034 | 0.004 | 3.82E-19 | 0.564 | 0.026 | 0.016 | 1.05E-01 | 2.19E-04 | 72 |
| 9 | 127405632 | rs4836984 | T | C | 0.493 | 0.034 | 0.004 | 3.92E-19 | 0.499 | -0.001 | 0.016 | 9.55E-01 | 2.19E-04 | 72 |
| 3 | 156795468 | rs13322435 | A | G | 0.581 | 0.036 | 0.004 | 4.13E-19 | 0.601 | 0.011 | 0.017 | 5.18E-01 | 2.46E-04 | 81 |
| 9 | 7174673 | rs913588 | A | G | 0.503 | -0.034 | 0.004 | 6.68E-19 | 0.519 | 0.002 | 0.016 | 8.84E-01 | 2.19E-04 | 72 |
| 12 | 49389320 | rs1054442* | A | C | 0.625 | 0.036 | 0.004 | 7.00E-19 | 0.623 | -0.034 | 0.016 | 3.38E-02 | 2.46E-04 | 81 |
| 21 | 40555561 | rs117143374 | T | C | 0.864 | -0.050 | 0.006 | 7.98E-19 | 0.862 | 0.015 | 0.023 | 5.27E-01 | 2.11E-04 | 70 |
| 8 | 87319950 | rs7465046 | T | C | 0.232 | -0.041 | 0.005 | 1.52E-18 | 0.240 | -0.013 | 0.019 | 4.78E-01 | 2.04E-04 | 67 |
| 9 | 92215638 | rs1571536* | T | C | 0.485 | 0.033 | 0.004 | 2.19E-18 | 0.479 | -0.020 | 0.016 | 2.15E-01 | 2.07E-04 | 68 |
| 2 | 157228255 | rs145438026 | T | C | 0.064 | -0.070 | 0.008 | 2.34E-18 | 0.063 | 0.035 | 0.032 | 2.75E-01 | 2.32E-04 | 76 |
| 8 | 3767623 | rs2688326 | T | C | 0.706 | -0.036 | 0.004 | 4.34E-18 | 0.698 | -0.008 | 0.017 | 6.26E-01 | 2.46E-04 | 81 |
| 4 | 104665972 | rs62342064 | T | C | 0.135 | 0.057 | 0.007 | 4.51E-18 | 0.144 | 0.003 | 0.023 | 8.90E-01 | 2.01E-04 | 66 |
| 4 | 28746246 | rs4340786 | A | T | 0.741 | 0.037 | 0.004 | 9.25E-18 | 0.743 | -0.014 | 0.018 | 4.36E-01 | 2.60E-04 | 86 |
| 6 | 100744134 | rs13199764 | T | C | 0.776 | 0.039 | 0.005 | 9.51E-18 | 0.775 | 0.013 | 0.019 | 4.87E-01 | 1.85E-04 | 61 |
| 9 | 83282402 | rs11534296 | A | G | 0.273 | -0.037 | 0.004 | 1.01E-17 | 0.263 | 0.044 | 0.018 | 1.38E-02 | 2.60E-04 | 86 |
| 11 | 78027488 | rs4945266 | A | G | 0.839 | -0.045 | 0.005 | 1.17E-17 | 0.845 | 0.010 | 0.022 | 6.32E-01 | 2.46E-04 | 81 |
| 7 | 74138121 | rs2267812* | A | C | 0.795 | 0.042 | 0.005 | 1.69E-17 | 0.790 | -0.011 | 0.019 | 5.72E-01 | 2.14E-04 | 70 |
| 8 | 78679087 | rs35485457 | T | G | 0.312 | -0.036 | 0.004 | 2.65E-17 | 0.326 | 0.003 | 0.017 | 8.47E-01 | 2.46E-04 | 81 |
| 21 | 37692507 | rs62229372 | T | C | 0.124 | 0.051 | 0.006 | 3.51E-17 | 0.125 | 0.035 | 0.024 | 1.43E-01 | 2.19E-04 | 72 |
| 13 | 74600274 | rs1925047 | A | C | 0.321 | -0.034 | 0.004 | 5.70E-17 | 0.317 | -0.019 | 0.017 | 2.74E-01 | 2.19E-04 | 72 |
| 11 | 27702383 | rs16917237* | T | G | 0.209 | 0.039 | 0.005 | 5.70E-17 | 0.203 | -0.023 | 0.020 | 2.47E-01 | 1.85E-04 | 61 |
| 6 | 151803754 | rs6933660 | A | C | 0.317 | -0.034 | 0.004 | 8.99E-17 | 0.316 | -0.020 | 0.017 | 2.37E-01 | 2.19E-04 | 72 |
| 8 | 140645701 | rs2542420 | C | G | 0.538 | 0.033 | 0.004 | 1.52E-16 | 0.529 | 0.015 | 0.016 | 3.43E-01 | 2.07E-04 | 68 |
| 8 | 4831685 | rs4875424 | T | C | 0.359 | -0.033 | 0.004 | 1.99E-16 | 0.365 | 0.009 | 0.017 | 5.78E-01 | 2.07E-04 | 68 |
| 17 | 6034754 | rs12603280 | A | G | 0.244 | -0.037 | 0.005 | 2.66E-16 | 0.245 | 0.015 | 0.019 | 4.08E-01 | 1.66E-04 | 55 |
| 2 | 142302503 | rs35935052 | T | G | 0.148 | 0.044 | 0.005 | 5.01E-16 | 0.151 | -0.019 | 0.022 | 3.82E-01 | 2.35E-04 | 77 |
| 16 | 19967668 | rs112991346* | T | C | 0.858 | -0.045 | 0.006 | 6.47E-16 | 0.857 | 0.054 | 0.023 | 1.89E-02 | 1.71E-04 | 56 |
| 20 | 37287102 | rs36093651 | T | C | 0.238 | 0.037 | 0.005 | 6.51E-16 | 0.238 | -0.001 | 0.019 | 9.39E-01 | 1.66E-04 | 55 |
| 1 | 102520898 | rs4561063 | T | G | 0.461 | 0.031 | 0.004 | 7.89E-16 | 0.469 | -0.009 | 0.016 | 5.64E-01 | 1.82E-04 | 60 |
| 19 | 7891767 | rs484353 | A | G | 0.539 | 0.032 | 0.004 | 8.28E-16 | 0.558 | 0.010 | 0.016 | 5.45E-01 | 1.94E-04 | 64 |
| 6 | 56859084 | rs9382676 | T | C | 0.777 | 0.037 | 0.005 | 8.77E-16 | 0.783 | -0.030 | 0.019 | 1.17E-01 | 1.66E-04 | 55 |
| 8 | 53877882 | rs16918378 | T | C | 0.877 | 0.048 | 0.006 | 9.08E-16 | 0.881 | -0.001 | 0.025 | 9.69E-01 | 1.94E-04 | 64 |
| 5 | 43134968 | rs7712046* | T | C | 0.696 | -0.033 | 0.004 | 9.42E-16 | 0.697 | 0.010 | 0.017 | 5.53E-01 | 2.07E-04 | 68 |
| 7 | 75142551 | rs187760798 | T | C | 0.884 | 0.058 | 0.007 | 1.10E-15 | 0.874 | -0.006 | 0.024 | 7.92E-01 | 2.08E-04 | 69 |
| 6 | 100983589 | rs12200565* | T | C | 0.539 | 0.032 | 0.004 | 1.11E-15 | 0.525 | -0.006 | 0.016 | 6.93E-01 | 1.94E-04 | 64 |
| 9 | 86764996 | rs13283567 | T | C | 0.152 | -0.044 | 0.006 | 1.31E-15 | 0.147 | -0.017 | 0.022 | 4.39E-01 | 1.63E-04 | 54 |
| 17 | 53209382 | rs2787487 | C | G | 0.604 | 0.031 | 0.004 | 1.62E-15 | 0.622 | 0.003 | 0.016 | 8.35E-01 | 1.82E-04 | 60 |
| 19 | 18829770 | rs11668587* | A | G | 0.665 | -0.033 | 0.004 | 1.86E-15 | 0.665 | 0.005 | 0.017 | 7.69E-01 | 2.07E-04 | 68 |
| 5 | 110876057 | rs247520 | T | C | 0.765 | 0.036 | 0.005 | 1.99E-15 | 0.771 | 0.000 | 0.019 | 9.93E-01 | 1.57E-04 | 52 |
| 11 | 115043574 | rs17564430* | T | G | 0.747 | -0.035 | 0.004 | 2.07E-15 | 0.743 | -0.014 | 0.018 | 4.35E-01 | 2.32E-04 | 76 |
| 3 | 127870060 | rs2461794 | A | G | 0.276 | 0.034 | 0.004 | 2.10E-15 | 0.266 | -0.017 | 0.018 | 3.47E-01 | 2.19E-04 | 72 |
| 1 | 21385436 | rs12125335 | T | C | 0.860 | -0.050 | 0.006 | 2.33E-15 | 0.861 | 0.021 | 0.023 | 3.68E-01 | 2.11E-04 | 70 |
| 6 | 76347020 | rs7753896 | A | G | 0.368 | 0.031 | 0.004 | 2.46E-15 | 0.374 | -0.009 | 0.016 | 5.68E-01 | 1.82E-04 | 60 |
| 14 | 93850179 | rs10143972* | T | C | 0.804 | -0.039 | 0.005 | 3.05E-15 | 0.808 | 0.010 | 0.020 | 6.04E-01 | 1.85E-04 | 61 |
| 7 | 41392815 | rs17171852 | A | C | 0.808 | -0.038 | 0.005 | 3.42E-15 | 0.804 | -0.029 | 0.020 | 1.40E-01 | 1.75E-04 | 58 |
| 5 | 168734867 | rs6864818 | T | C | 0.211 | 0.036 | 0.005 | 4.67E-15 | 0.202 | 0.030 | 0.020 | 1.30E-01 | 1.57E-04 | 52 |
| 11 | 30317733 | rs11031040 | T | G | 0.838 | -0.040 | 0.005 | 5.10E-15 | 0.840 | 0.031 | 0.022 | 1.55E-01 | 1.94E-04 | 64 |
| 3 | 24206463 | rs73035994 | T | C | 0.972 | -0.091 | 0.012 | 5.22E-15 | 0.973 | -0.088 | 0.048 | 6.60E-02 | 1.75E-04 | 58 |
| 11 | 46064974 | rs953230 | A | G | 0.708 | 0.033 | 0.004 | 5.52E-15 | 0.712 | -0.035 | 0.018 | 4.54E-02 | 2.07E-04 | 68 |
| 17 | 79217478 | rs2659007 | A | G | 0.465 | -0.031 | 0.004 | 6.23E-15 | 0.449 | 0.019 | 0.016 | 2.28E-01 | 1.82E-04 | 60 |
| 13 | 40238492 | rs9548873 | T | C | 0.663 | -0.031 | 0.004 | 7.50E-15 | 0.663 | 0.002 | 0.017 | 9.18E-01 | 1.82E-04 | 60 |
| 10 | 121708929 | rs12571664 | T | C | 0.800 | 0.037 | 0.005 | 1.85E-14 | 0.815 | -0.017 | 0.020 | 4.04E-01 | 1.66E-04 | 55 |
| 3 | 132610752 | rs6439371 | A | G | 0.648 | -0.030 | 0.004 | 2.35E-14 | 0.647 | -0.018 | 0.017 | 2.85E-01 | 1.71E-04 | 56 |
| 9 | 111809295 | rs11792861 | A | C | 0.709 | 0.032 | 0.004 | 2.51E-14 | 0.707 | 0.004 | 0.017 | 8.35E-01 | 1.94E-04 | 64 |
| 2 | 203168235 | rs16841867 | C | G | 0.885 | 0.046 | 0.006 | 2.56E-14 | 0.886 | -0.014 | 0.025 | 5.63E-01 | 1.78E-04 | 59 |
| 1 | 204158132 | rs11240695 | A | C | 0.251 | -0.033 | 0.004 | 3.06E-14 | 0.248 | 0.031 | 0.018 | 9.13E-02 | 2.07E-04 | 68 |
| 11 | 206089 | rs3782120 | A | G | 0.258 | 0.033 | 0.004 | 3.69E-14 | 0.250 | -0.014 | 0.018 | 4.34E-01 | 2.07E-04 | 68 |
| 1 | 98375448 | rs11165924 | A | G | 0.677 | 0.031 | 0.004 | 4.83E-14 | 0.667 | 0.025 | 0.017 | 1.31E-01 | 1.82E-04 | 60 |
| 12 | 17126283 | rs77530428 | A | G | 0.983 | -0.124 | 0.017 | 6.25E-14 | 0.984 | 0.037 | 0.064 | 5.67E-01 | 1.62E-04 | 53 |
| 3 | 51358019 | rs6445624 | A | G | 0.147 | 0.042 | 0.006 | 6.41E-14 | 0.138 | 0.033 | 0.023 | 1.42E-01 | 1.49E-04 | 49 |
| 3 | 88221517 | rs4859001* | T | C | 0.148 | 0.045 | 0.006 | 6.64E-14 | 0.155 | 0.023 | 0.022 | 2.79E-01 | 1.71E-04 | 56 |
| 6 | 28441634 | rs12663002 | T | C | 0.129 | 0.043 | 0.006 | 6.89E-14 | 0.122 | -0.009 | 0.024 | 6.98E-01 | 1.56E-04 | 51 |
| 19 | 1828948 | rs3746037* | A | C | 0.787 | 0.036 | 0.005 | 1.01E-13 | 0.793 | 0.045 | 0.020 | 2.13E-02 | 1.57E-04 | 52 |
| 9 | 1711210 | rs552491 | A | G | 0.638 | -0.029 | 0.004 | 1.11E-13 | 0.627 | -0.007 | 0.016 | 6.89E-01 | 1.60E-04 | 53 |
| 4 | 3266860 | rs2108753 | T | C | 0.565 | 0.028 | 0.004 | 1.20E-13 | 0.570 | 0.015 | 0.016 | 3.58E-01 | 1.49E-04 | 49 |
| 6 | 108867031 | rs6911407* | A | C | 0.376 | 0.029 | 0.004 | 1.22E-13 | 0.368 | -0.016 | 0.016 | 3.14E-01 | 1.60E-04 | 53 |
| 7 | 121955981 | rs10237306* | T | G | 0.390 | 0.030 | 0.004 | 1.34E-13 | 0.383 | 0.034 | 0.016 | 3.73E-02 | 1.71E-04 | 56 |
| 9 | 92515514 | rs9330454 | A | G | 0.430 | -0.031 | 0.004 | 1.44E-13 | 0.433 | 0.013 | 0.016 | 4.30E-01 | 1.82E-04 | 60 |
| 13 | 112285043 | rs74499585 | A | G | 0.087 | 0.058 | 0.008 | 1.55E-13 | 0.085 | 0.072 | 0.028 | 9.61E-03 | 1.60E-04 | 53 |
| 11 | 28033473 | rs11606190 | A | G | 0.146 | 0.041 | 0.006 | 1.61E-13 | 0.147 | -0.078 | 0.023 | 5.80E-04 | 1.42E-04 | 47 |
| 3 | 184030827 | rs7649124 | C | G | 0.757 | 0.033 | 0.005 | 2.00E-13 | 0.752 | 0.021 | 0.018 | 2.58E-01 | 1.32E-04 | 43 |
| 20 | 20348962 | rs111558392 | T | C | 0.153 | -0.040 | 0.006 | 2.09E-13 | 0.156 | -0.003 | 0.022 | 8.88E-01 | 1.35E-04 | 44 |
| 15 | 83406228 | rs1971554 | T | C | 0.265 | 0.032 | 0.004 | 2.35E-13 | 0.258 | -0.014 | 0.018 | 4.56E-01 | 1.94E-04 | 64 |
| 9 | 73798371 | rs1329767 | A | C | 0.349 | -0.029 | 0.004 | 2.43E-13 | 0.353 | 0.028 | 0.016 | 8.42E-02 | 1.60E-04 | 53 |
| 6 | 77173780 | rs7757654 | T | C | 0.296 | -0.031 | 0.004 | 2.51E-13 | 0.305 | 0.035 | 0.017 | 4.32E-02 | 1.82E-04 | 60 |
| 22 | 22273242 | rs151680 | T | C | 0.524 | 0.028 | 0.004 | 2.52E-13 | 0.527 | 0.000 | 0.016 | 9.87E-01 | 1.49E-04 | 49 |
| 17 | 78739672 | rs66508321 | A | G | 0.324 | -0.030 | 0.004 | 2.84E-13 | 0.326 | 0.033 | 0.017 | 5.10E-02 | 1.71E-04 | 56 |
| 5 | 137761555 | rs3815212 | T | C | 0.783 | 0.034 | 0.005 | 3.21E-13 | 0.798 | -0.025 | 0.020 | 2.02E-01 | 1.40E-04 | 46 |
| 10 | 126861278 | rs9422857 | C | G | 0.506 | -0.028 | 0.004 | 3.67E-13 | 0.499 | -0.037 | 0.016 | 1.89E-02 | 1.49E-04 | 49 |
| 2 | 61367664 | rs10205969 | T | C | 0.145 | -0.039 | 0.005 | 5.62E-13 | 0.141 | -0.026 | 0.023 | 2.65E-01 | 1.85E-04 | 61 |
| 6 | 50930848 | rs79541760* | A | T | 0.831 | 0.037 | 0.005 | 6.98E-13 | 0.837 | -0.010 | 0.022 | 6.29E-01 | 1.66E-04 | 55 |
| 11 | 43608835 | rs1023955* | T | G | 0.398 | -0.028 | 0.004 | 7.36E-13 | 0.404 | 0.010 | 0.016 | 5.41E-01 | 1.49E-04 | 49 |
| 9 | 11813745 | rs1601615 | T | C | 0.396 | -0.028 | 0.004 | 7.57E-13 | 0.386 | 0.012 | 0.016 | 4.73E-01 | 1.49E-04 | 49 |
| 15 | 24824016 | rs8040272 | A | G | 0.868 | 0.044 | 0.006 | 7.79E-13 | 0.875 | -0.001 | 0.024 | 9.63E-01 | 1.63E-04 | 54 |
| 22 | 31293700 | rs5753377 | A | G | 0.287 | -0.031 | 0.004 | 8.04E-13 | 0.276 | 0.003 | 0.018 | 8.69E-01 | 1.82E-04 | 60 |
| 15 | 64537300 | rs72756954 | C | G | 0.938 | 0.058 | 0.008 | 8.45E-13 | 0.938 | -0.019 | 0.033 | 5.58E-01 | 1.60E-04 | 53 |
| 12 | 47876942 | rs7971408 | T | C | 0.106 | 0.045 | 0.006 | 9.36E-13 | 0.108 | -0.007 | 0.026 | 7.81E-01 | 1.71E-04 | 56 |
| 15 | 23810843 | rs184950120 | T | C | 0.005 | -0.396 | 0.056 | 9.78E-13 | 0.003 | -0.218 | 0.187 | 2.29E-01 | 1.52E-04 | 50 |
| 10 | 123676662 | rs7077302 | C | G | 0.085 | 0.050 | 0.007 | 1.08E-12 | 0.080 | 0.004 | 0.029 | 9.04E-01 | 1.55E-04 | 51 |
| 11 | 119059404 | rs1815811 | A | G | 0.536 | -0.027 | 0.004 | 1.65E-12 | 0.530 | 0.021 | 0.016 | 1.87E-01 | 1.38E-04 | 45 |
| 5 | 111130474 | rs1566385 | A | G | 0.942 | 0.060 | 0.008 | 1.81E-12 | 0.937 | -0.049 | 0.032 | 1.34E-01 | 1.71E-04 | 56 |
| 8 | 53934144 | rs56409371 | A | G | 0.784 | -0.035 | 0.005 | 1.95E-12 | 0.792 | 0.004 | 0.020 | 8.46E-01 | 1.49E-04 | 49 |
| 17 | 43123625 | rs59246405 | T | C | 0.405 | 0.027 | 0.004 | 2.03E-12 | 0.415 | -0.034 | 0.016 | 3.44E-02 | 1.38E-04 | 45 |
| 1 | 150758727 | rs6661100 | T | C | 0.092 | 0.047 | 0.007 | 2.18E-12 | 0.080 | -0.032 | 0.029 | 2.75E-01 | 1.37E-04 | 45 |
| 6 | 29740548 | rs62391851 | A | G | 0.059 | -0.060 | 0.009 | 2.44E-12 | 0.060 | 0.005 | 0.033 | 8.85E-01 | 1.35E-04 | 44 |
| 2 | 153556801 | rs6434162 | A | G | 0.824 | -0.036 | 0.005 | 2.51E-12 | 0.826 | -0.020 | 0.021 | 3.27E-01 | 1.57E-04 | 52 |
| 5 | 52909927 | rs813301 | T | C | 0.626 | 0.027 | 0.004 | 2.84E-12 | 0.630 | 0.011 | 0.016 | 5.06E-01 | 1.38E-04 | 45 |
| 15 | 47925066 | rs1435753* | T | C | 0.647 | -0.028 | 0.004 | 2.91E-12 | 0.650 | 0.027 | 0.017 | 9.75E-02 | 1.49E-04 | 49 |
| 13 | 59833519 | rs4886140 | A | G | 0.333 | 0.028 | 0.004 | 3.10E-12 | 0.332 | 0.007 | 0.017 | 6.68E-01 | 1.49E-04 | 49 |
| 3 | 18442437 | rs9867904 | C | G | 0.387 | -0.028 | 0.004 | 3.42E-12 | 0.406 | 0.002 | 0.016 | 8.99E-01 | 1.49E-04 | 49 |
| 16 | 30102802 | rs3809624* | T | C | 0.677 | -0.030 | 0.004 | 3.64E-12 | 0.677 | 0.047 | 0.017 | 6.11E-03 | 1.71E-04 | 56 |
| 11 | 77555824 | rs7115444 | T | C | 0.209 | 0.033 | 0.005 | 3.82E-12 | 0.202 | 0.014 | 0.020 | 4.87E-01 | 1.32E-04 | 43 |
| 7 | 132729814 | rs12707076 | C | G | 0.384 | 0.027 | 0.004 | 4.88E-12 | 0.371 | -0.002 | 0.016 | 9.07E-01 | 1.38E-04 | 45 |
| 9 | 109554196 | rs10978641 | A | T | 0.746 | -0.032 | 0.005 | 5.10E-12 | 0.735 | -0.024 | 0.018 | 1.83E-01 | 1.24E-04 | 41 |
| 1 | 205717823 | rs4951261 | A | C | 0.614 | 0.027 | 0.004 | 5.36E-12 | 0.605 | -0.002 | 0.016 | 9.00E-01 | 1.38E-04 | 45 |
| 7 | 122160742 | rs11767400 | A | C | 0.296 | 0.029 | 0.004 | 5.41E-12 | 0.305 | 0.002 | 0.017 | 8.94E-01 | 1.60E-04 | 53 |
| 20 | 54823805 | rs3746619 | A | C | 0.086 | 0.048 | 0.007 | 5.52E-12 | 0.084 | 0.002 | 0.029 | 9.39E-01 | 1.43E-04 | 47 |
| 20 | 33447915 | rs2295094 | A | G | 0.159 | 0.036 | 0.005 | 5.86E-12 | 0.172 | 0.040 | 0.021 | 5.52E-02 | 1.57E-04 | 52 |
| 5 | 180656734 | rs2770957 | C | G | 0.777 | 0.032 | 0.005 | 5.90E-12 | 0.785 | 0.005 | 0.019 | 7.83E-01 | 1.24E-04 | 41 |
| 11 | 16596152 | rs4359170 | A | T | 0.673 | 0.028 | 0.004 | 5.99E-12 | 0.678 | -0.005 | 0.017 | 7.81E-01 | 1.49E-04 | 49 |
| 10 | 102686073 | rs72842141 | A | T | 0.943 | -0.062 | 0.009 | 6.12E-12 | 0.937 | 0.050 | 0.034 | 1.38E-01 | 1.44E-04 | 47 |
| 19 | 34309532 | rs29941* | A | G | 0.323 | 0.028 | 0.004 | 7.85E-12 | 0.328 | -0.018 | 0.017 | 2.82E-01 | 1.49E-04 | 49 |
| 2 | 61685826 | rs12467441 | T | C | 0.874 | -0.041 | 0.006 | 8.99E-12 | 0.874 | -0.032 | 0.024 | 1.78E-01 | 1.42E-04 | 47 |
| 2 | 184291116 | rs842567 | A | C | 0.794 | -0.034 | 0.005 | 9.38E-12 | 0.801 | 0.003 | 0.020 | 8.80E-01 | 1.40E-04 | 46 |
| 3 | 114574749 | rs9834893 | C | G | 0.929 | -0.051 | 0.007 | 9.41E-12 | 0.937 | -0.034 | 0.032 | 2.89E-01 | 1.61E-04 | 53 |
| 8 | 25280800 | rs6185 | C | G | 0.730 | -0.030 | 0.004 | 9.48E-12 | 0.750 | 0.022 | 0.018 | 2.32E-01 | 1.71E-04 | 56 |
| 9 | 96276910 | rs10992769 | C | G | 0.709 | 0.029 | 0.004 | 1.11E-11 | 0.713 | 0.022 | 0.018 | 2.04E-01 | 1.60E-04 | 53 |
| 5 | 156715068 | rs437836 | T | C | 0.167 | 0.035 | 0.005 | 1.15E-11 | 0.164 | -0.014 | 0.021 | 5.19E-01 | 1.49E-04 | 49 |
| 12 | 108618630 | rs3764002* | T | C | 0.265 | -0.030 | 0.005 | 1.21E-11 | 0.263 | -0.021 | 0.018 | 2.43E-01 | 1.09E-04 | 36 |
| 17 | 77796437 | rs7218751 | A | G | 0.811 | 0.033 | 0.005 | 1.37E-11 | 0.808 | -0.012 | 0.020 | 5.50E-01 | 1.32E-04 | 43 |
| 3 | 172121443 | rs582780 | A | G | 0.582 | 0.027 | 0.004 | 1.41E-11 | 0.577 | 0.017 | 0.016 | 2.93E-01 | 1.38E-04 | 45 |
| 16 | 20380004 | rs4780885* | C | G | 0.493 | 0.026 | 0.004 | 1.52E-11 | 0.499 | -0.025 | 0.016 | 1.18E-01 | 1.28E-04 | 42 |
| 4 | 177465182 | rs13120031 | T | C | 0.324 | 0.027 | 0.004 | 1.57E-11 | 0.314 | 0.012 | 0.017 | 4.84E-01 | 1.38E-04 | 45 |
| 4 | 104774698 | rs115260227 | A | G | 0.987 | -0.162 | 0.024 | 1.90E-11 | 0.985 | 0.006 | 0.070 | 9.33E-01 | 1.38E-04 | 45 |
| 20 | 54830983 | rs13043968 | A | C | 0.111 | -0.041 | 0.006 | 1.92E-11 | 0.108 | 0.027 | 0.026 | 2.92E-01 | 1.42E-04 | 47 |
| 19 | 31051857 | rs56367141* | A | C | 0.131 | -0.040 | 0.006 | 2.11E-11 | 0.143 | -0.023 | 0.023 | 3.13E-01 | 1.35E-04 | 44 |
| 4 | 106066293 | rs17035311 | A | C | 0.854 | 0.036 | 0.005 | 2.26E-11 | 0.862 | -0.044 | 0.023 | 5.52E-02 | 1.57E-04 | 52 |
| 11 | 86716236 | rs7108556 | T | C | 0.779 | 0.031 | 0.005 | 2.62E-11 | 0.780 | -0.010 | 0.019 | 5.95E-01 | 1.17E-04 | 39 |
| 10 | 120833948 | rs10400136 | A | G | 0.563 | -0.026 | 0.004 | 2.73E-11 | 0.547 | 0.020 | 0.016 | 2.02E-01 | 1.28E-04 | 42 |
| 7 | 138874416 | rs13233916* | C | G | 0.907 | -0.050 | 0.008 | 3.11E-11 | 0.910 | 0.004 | 0.028 | 8.74E-01 | 1.19E-04 | 39 |
| 11 | 84780098 | rs4402316 | C | G | 0.241 | 0.031 | 0.005 | 3.22E-11 | 0.264 | -0.004 | 0.018 | 8.41E-01 | 1.17E-04 | 39 |
| 11 | 94085099 | rs113557523 | T | C | 0.086 | -0.045 | 0.007 | 3.75E-11 | 0.079 | -0.056 | 0.030 | 5.67E-02 | 1.25E-04 | 41 |
| 15 | 24183428 | rs4778356 | A | G | 0.865 | 0.039 | 0.006 | 4.01E-11 | 0.859 | 0.009 | 0.023 | 6.87E-01 | 1.28E-04 | 42 |
| 8 | 132071766 | rs7826872 | T | C | 0.447 | 0.026 | 0.004 | 4.26E-11 | 0.444 | 0.000 | 0.016 | 9.97E-01 | 1.28E-04 | 42 |
| 20 | 19682834 | rs1535252 | T | C | 0.455 | -0.025 | 0.004 | 4.82E-11 | 0.443 | -0.014 | 0.016 | 3.66E-01 | 1.19E-04 | 39 |
| 4 | 132621869 | rs62316795 | A | C | 0.193 | 0.035 | 0.005 | 4.98E-11 | 0.198 | 0.007 | 0.020 | 7.26E-01 | 1.49E-04 | 49 |
| 14 | 97769834 | rs10145469 | A | C | 0.051 | -0.061 | 0.009 | 5.56E-11 | 0.051 | -0.054 | 0.037 | 1.43E-01 | 1.39E-04 | 46 |
| 12 | 24579079 | rs10842343 | A | T | 0.604 | -0.026 | 0.004 | 5.78E-11 | 0.609 | 0.003 | 0.016 | 8.70E-01 | 1.28E-04 | 42 |
| 5 | 167370263 | rs9647570 | T | G | 0.854 | -0.036 | 0.006 | 6.26E-11 | 0.859 | 0.015 | 0.023 | 5.23E-01 | 1.09E-04 | 36 |
| 10 | 118696266 | rs4751614* | A | T | 0.766 | 0.030 | 0.005 | 6.86E-11 | 0.767 | -0.002 | 0.019 | 9.21E-01 | 1.09E-04 | 36 |
| 19 | 18346228 | rs12460047* | A | G | 0.283 | -0.028 | 0.004 | 6.93E-11 | 0.281 | -0.023 | 0.018 | 1.98E-01 | 1.49E-04 | 49 |
| 1 | 8436802 | rs6678140 | T | C | 0.673 | -0.027 | 0.004 | 7.54E-11 | 0.658 | -0.007 | 0.017 | 6.58E-01 | 1.38E-04 | 45 |
| 5 | 153541904 | rs1428120* | T | G | 0.572 | 0.025 | 0.004 | 7.72E-11 | 0.572 | -0.021 | 0.016 | 1.99E-01 | 1.19E-04 | 39 |
| 6 | 52946320 | rs222440 | T | C | 0.186 | -0.033 | 0.005 | 9.73E-11 | 0.179 | 0.014 | 0.021 | 4.97E-01 | 1.32E-04 | 43 |
| 3 | 49254427 | rs6803264 | T | C | 0.230 | 0.030 | 0.005 | 9.94E-11 | 0.218 | 0.004 | 0.019 | 8.16E-01 | 1.09E-04 | 36 |
| 11 | 65473798 | rs10750766 | A | C | 0.709 | -0.028 | 0.004 | 9.96E-11 | 0.709 | 0.028 | 0.017 | 1.07E-01 | 1.49E-04 | 49 |
| 9 | 77273910 | rs35436838 | T | G | 0.958 | -0.068 | 0.011 | 1.27E-10 | 0.953 | 0.019 | 0.038 | 6.20E-01 | 1.16E-04 | 38 |
| 20 | 62799680 | rs443252 | T | C | 0.050 | 0.059 | 0.009 | 1.29E-10 | 0.055 | -0.117 | 0.037 | 1.15E-03 | 1.30E-04 | 43 |
| 19 | 4980864 | rs169080 | T | C | 0.354 | -0.026 | 0.004 | 1.33E-10 | 0.360 | 0.003 | 0.016 | 8.33E-01 | 1.28E-04 | 42 |
| 11 | 46539110 | rs68002803 | T | C | 0.677 | 0.027 | 0.004 | 1.41E-10 | 0.679 | -0.016 | 0.017 | 3.56E-01 | 1.38E-04 | 45 |
| 6 | 128364709 | rs4327718 | A | G | 0.828 | -0.033 | 0.005 | 2.04E-10 | 0.821 | -0.018 | 0.021 | 3.79E-01 | 1.32E-04 | 43 |
| 3 | 137128815 | rs6439713 | A | C | 0.315 | 0.026 | 0.004 | 2.13E-10 | 0.314 | 0.018 | 0.017 | 2.78E-01 | 1.28E-04 | 42 |
| 9 | 10283451 | rs10959016 | A | G | 0.206 | -0.031 | 0.005 | 2.21E-10 | 0.204 | -0.001 | 0.020 | 9.77E-01 | 1.17E-04 | 39 |
| 5 | 139384490 | rs975642 | T | C | 0.494 | -0.025 | 0.004 | 2.51E-10 | 0.486 | -0.022 | 0.016 | 1.65E-01 | 1.19E-04 | 39 |
| 6 | 18559687 | rs6927679* | T | C | 0.718 | 0.027 | 0.004 | 2.52E-10 | 0.719 | -0.019 | 0.017 | 2.78E-01 | 1.38E-04 | 45 |
| 2 | 65279414 | rs2723065 | A | G | 0.613 | -0.025 | 0.004 | 2.59E-10 | 0.623 | 0.002 | 0.016 | 9.25E-01 | 1.19E-04 | 39 |
| 7 | 99701176 | rs999885 | A | G | 0.516 | 0.024 | 0.004 | 2.79E-10 | 0.513 | -0.008 | 0.016 | 5.92E-01 | 1.09E-04 | 36 |
| 5 | 64020316 | rs80170948 | T | G | 0.956 | -0.069 | 0.011 | 2.80E-10 | 0.955 | -0.028 | 0.040 | 4.85E-01 | 1.19E-04 | 39 |
| 5 | 41994067 | rs62361685 | T | C | 0.942 | 0.054 | 0.009 | 3.09E-10 | 0.938 | 0.040 | 0.033 | 2.30E-01 | 1.09E-04 | 36 |
| 1 | 213451958 | rs12040029 | T | C | 0.128 | -0.039 | 0.006 | 4.04E-10 | 0.123 | 0.011 | 0.025 | 6.59E-01 | 1.28E-04 | 42 |
| 8 | 77653945 | rs11786868 | C | G | 0.837 | 0.032 | 0.005 | 4.27E-10 | 0.845 | 0.029 | 0.022 | 1.83E-01 | 1.24E-04 | 41 |
| 5 | 138281261 | rs6878910 | A | G | 0.153 | 0.038 | 0.006 | 4.31E-10 | 0.152 | 0.049 | 0.022 | 2.49E-02 | 1.22E-04 | 40 |
| 9 | 11130009 | rs10959552 | A | G | 0.885 | -0.037 | 0.006 | 4.50E-10 | 0.881 | -0.009 | 0.024 | 7.05E-01 | 1.15E-04 | 38 |
| 16 | 3627358 | rs758747* | T | C | 0.276 | -0.027 | 0.004 | 5.56E-10 | 0.281 | -0.007 | 0.018 | 7.04E-01 | 1.38E-04 | 45 |
| 9 | 80513323 | rs2378100* | T | C | 0.570 | -0.024 | 0.004 | 6.35E-10 | 0.572 | 0.015 | 0.016 | 3.44E-01 | 1.09E-04 | 36 |
| 17 | 79446015 | rs12937034 | A | G | 0.614 | -0.025 | 0.004 | 6.43E-10 | 0.600 | -0.003 | 0.016 | 8.65E-01 | 1.19E-04 | 39 |
| 3 | 52719816 | rs10933 | T | C | 0.442 | -0.024 | 0.004 | 6.72E-10 | 0.454 | -0.015 | 0.016 | 3.39E-01 | 1.09E-04 | 36 |
| 5 | 107316227 | rs2916578 | A | G | 0.585 | -0.025 | 0.004 | 6.99E-10 | 0.558 | -0.001 | 0.016 | 9.58E-01 | 1.19E-04 | 39 |
| 10 | 134294398 | rs4576738 | A | G | 0.446 | 0.027 | 0.004 | 7.20E-10 | 0.495 | -0.004 | 0.016 | 8.19E-01 | 1.38E-04 | 45 |
| 1 | 82562929 | rs10782777 | A | G | 0.640 | -0.025 | 0.004 | 8.40E-10 | 0.637 | 0.010 | 0.017 | 5.52E-01 | 1.19E-04 | 39 |
| 16 | 15542199 | rs153793 | A | G | 0.520 | -0.023 | 0.004 | 9.27E-10 | 0.527 | -0.032 | 0.016 | 4.59E-02 | 1.00E-04 | 33 |
| 15 | 54364552 | rs11852771 | A | G | 0.385 | 0.024 | 0.004 | 9.69E-10 | 0.382 | 0.033 | 0.016 | 4.28E-02 | 1.09E-04 | 36 |
| 1 | 41456689 | rs11209331 | T | C | 0.571 | 0.024 | 0.004 | 9.83E-10 | 0.568 | 0.006 | 0.016 | 6.96E-01 | 1.09E-04 | 36 |
| 15 | 74336633 | rs5742915 | T | C | 0.546 | -0.023 | 0.004 | 1.02E-09 | 0.540 | 0.007 | 0.016 | 6.64E-01 | 1.00E-04 | 33 |
| 15 | 41494364 | rs4924538 | A | T | 0.513 | -0.026 | 0.004 | 1.03E-09 | 0.471 | -0.010 | 0.016 | 5.31E-01 | 1.28E-04 | 42 |
| 6 | 136228617 | rs78928932 | T | C | 0.931 | -0.055 | 0.009 | 1.06E-09 | 0.924 | 0.041 | 0.032 | 1.98E-01 | 1.13E-04 | 37 |
| 17 | 77951023 | rs59652033 | T | C | 0.348 | -0.026 | 0.004 | 1.16E-09 | 0.348 | 0.006 | 0.017 | 7.24E-01 | 1.28E-04 | 42 |
| 17 | 7774047 | rs55680968 | A | G | 0.928 | -0.046 | 0.008 | 1.17E-09 | 0.924 | -0.012 | 0.030 | 6.87E-01 | 1.00E-04 | 33 |
| 10 | 2697434 | rs7912468 | T | C | 0.578 | -0.024 | 0.004 | 1.20E-09 | 0.579 | 0.004 | 0.016 | 8.08E-01 | 1.09E-04 | 36 |
| 10 | 51056858 | rs61846901 | T | C | 0.311 | -0.026 | 0.004 | 1.21E-09 | 0.313 | 0.015 | 0.017 | 3.75E-01 | 1.28E-04 | 42 |
| 7 | 94186064 | rs15671 | A | C | 0.421 | -0.023 | 0.004 | 1.25E-09 | 0.417 | 0.022 | 0.016 | 1.73E-01 | 1.00E-04 | 33 |
| 16 | 24761970 | rs113245667 | C | G | 0.958 | -0.062 | 0.010 | 1.26E-09 | 0.959 | 0.002 | 0.040 | 9.64E-01 | 1.17E-04 | 39 |
| 2 | 69704941 | rs2312205 | A | G | 0.822 | 0.030 | 0.005 | 1.26E-09 | 0.816 | 0.009 | 0.020 | 6.57E-01 | 1.09E-04 | 36 |
| 10 | 104871361 | rs2066323 | A | G | 0.602 | -0.024 | 0.004 | 1.31E-09 | 0.612 | -0.029 | 0.016 | 6.98E-02 | 1.09E-04 | 36 |
| 19 | 50334895 | rs4801809 | T | C | 0.096 | -0.044 | 0.007 | 1.33E-09 | 0.090 | 0.072 | 0.029 | 1.25E-02 | 1.20E-04 | 40 |
| 1 | 163018934 | rs2661339 | T | G | 0.054 | 0.053 | 0.009 | 1.34E-09 | 0.047 | 0.023 | 0.037 | 5.33E-01 | 1.05E-04 | 35 |
| 22 | 45745229 | rs9614460 | T | G | 0.678 | -0.025 | 0.004 | 1.38E-09 | 0.681 | 0.040 | 0.017 | 1.87E-02 | 1.19E-04 | 39 |
| 4 | 45910674 | rs4588499 | A | G | 0.494 | -0.024 | 0.004 | 1.47E-09 | 0.503 | 0.003 | 0.016 | 8.65E-01 | 1.09E-04 | 36 |
| 1 | 65972550 | rs7516763 | A | C | 0.469 | 0.023 | 0.004 | 1.49E-09 | 0.467 | 0.000 | 0.016 | 9.81E-01 | 1.00E-04 | 33 |
| 10 | 63660689 | rs6415872 | A | G | 0.493 | 0.024 | 0.004 | 1.52E-09 | 0.496 | -0.004 | 0.016 | 8.04E-01 | 1.09E-04 | 36 |
| 12 | 115107376 | rs474463 | T | C | 0.219 | -0.029 | 0.005 | 1.65E-09 | 0.230 | -0.017 | 0.019 | 3.58E-01 | 1.02E-04 | 34 |
| 5 | 167947996 | rs4976623 | C | G | 0.189 | 0.030 | 0.005 | 1.71E-09 | 0.187 | 0.007 | 0.021 | 7.15E-01 | 1.09E-04 | 36 |
| 8 | 105329549 | rs2441873 | T | G | 0.411 | 0.024 | 0.004 | 1.74E-09 | 0.410 | -0.005 | 0.016 | 7.68E-01 | 1.09E-04 | 36 |
| 5 | 167404411 | rs2546959 | T | G | 0.827 | 0.032 | 0.005 | 1.82E-09 | 0.835 | -0.017 | 0.022 | 4.39E-01 | 1.24E-04 | 41 |
| 5 | 179034260 | rs4701140 | A | G | 0.512 | 0.024 | 0.004 | 1.95E-09 | 0.490 | 0.001 | 0.016 | 9.31E-01 | 1.09E-04 | 36 |
| 12 | 111600134 | rs11065822 | T | G | 0.373 | 0.026 | 0.004 | 1.96E-09 | 0.357 | 0.030 | 0.017 | 7.61E-02 | 1.28E-04 | 42 |
| 10 | 103754188 | rs59543819 | T | C | 0.705 | -0.025 | 0.004 | 1.97E-09 | 0.716 | 0.026 | 0.018 | 1.38E-01 | 1.19E-04 | 39 |
| 19 | 13104027 | rs10422323 | A | G | 0.121 | 0.037 | 0.006 | 2.01E-09 | 0.126 | 0.000 | 0.024 | 9.99E-01 | 1.15E-04 | 38 |
| 11 | 63593219 | rs10897450 | C | G | 0.530 | 0.023 | 0.004 | 2.18E-09 | 0.535 | 0.011 | 0.016 | 4.82E-01 | 1.00E-04 | 33 |
| 10 | 13541008 | rs10906395 | T | C | 0.611 | -0.023 | 0.004 | 2.28E-09 | 0.591 | 0.031 | 0.016 | 5.76E-02 | 1.00E-04 | 33 |
| 9 | 120730928 | rs2780243* | T | C | 0.565 | -0.023 | 0.004 | 2.31E-09 | 0.559 | 0.008 | 0.016 | 6.05E-01 | 1.00E-04 | 33 |
| 1 | 174031021 | rs61826838 | T | C | 0.827 | -0.032 | 0.005 | 2.34E-09 | 0.846 | -0.011 | 0.023 | 6.32E-01 | 1.24E-04 | 41 |
| 5 | 110503301 | rs654354 | A | T | 0.383 | -0.023 | 0.004 | 2.36E-09 | 0.390 | -0.021 | 0.016 | 2.00E-01 | 1.00E-04 | 33 |
| 8 | 34902952 | rs13278754 | C | G | 0.283 | -0.025 | 0.004 | 2.40E-09 | 0.282 | -0.025 | 0.018 | 1.50E-01 | 1.19E-04 | 39 |
| 1 | 150423577 | rs61817552 | A | G | 0.211 | -0.029 | 0.005 | 2.42E-09 | 0.220 | 0.024 | 0.019 | 2.03E-01 | 1.02E-04 | 34 |
| 9 | 22819576 | rs7849973 | C | G | 0.655 | 0.024 | 0.004 | 2.82E-09 | 0.659 | 0.020 | 0.017 | 2.26E-01 | 1.09E-04 | 36 |
| 1 | 162895515 | rs2343507 | A | C | 0.588 | 0.023 | 0.004 | 2.88E-09 | 0.601 | 0.010 | 0.016 | 5.52E-01 | 1.00E-04 | 33 |
| 1 | 33228197 | rs360495 | A | T | 0.087 | 0.043 | 0.007 | 2.89E-09 | 0.086 | -0.031 | 0.029 | 2.76E-01 | 1.15E-04 | 38 |
| 1 | 91196099 | rs7517629 | A | G | 0.471 | -0.024 | 0.004 | 3.10E-09 | 0.454 | -0.006 | 0.016 | 7.19E-01 | 1.09E-04 | 36 |
| 6 | 148285329 | rs6911527 | T | C | 0.228 | 0.027 | 0.005 | 3.18E-09 | 0.231 | -0.004 | 0.019 | 8.31E-01 | 8.85E-05 | 29 |
| 22 | 39157755 | rs4303811 | A | G | 0.124 | -0.035 | 0.006 | 3.19E-09 | 0.118 | -0.020 | 0.025 | 4.31E-01 | 1.03E-04 | 34 |
| 10 | 88081438 | rs77532868 | T | C | 0.045 | 0.057 | 0.010 | 3.31E-09 | 0.054 | 0.016 | 0.035 | 6.45E-01 | 9.86E-05 | 32 |
| 15 | 51507610 | rs28757192 | T | C | 0.034 | -0.063 | 0.011 | 3.50E-09 | 0.034 | 0.022 | 0.044 | 6.16E-01 | 9.96E-05 | 33 |
| 5 | 95630705 | rs17085593* | C | G | 0.684 | 0.025 | 0.004 | 3.53E-09 | 0.687 | -0.014 | 0.017 | 4.21E-01 | 1.19E-04 | 39 |
| 2 | 44952254 | rs17390720 | C | G | 0.735 | 0.026 | 0.004 | 3.70E-09 | 0.735 | 0.015 | 0.018 | 4.10E-01 | 1.28E-04 | 42 |
| 12 | 56435929 | rs1131017* | C | G | 0.417 | 0.023 | 0.004 | 3.75E-09 | 0.431 | 0.012 | 0.016 | 4.44E-01 | 1.00E-04 | 33 |
| 20 | 21485806 | rs4813429 | T | C | 0.831 | 0.031 | 0.005 | 4.17E-09 | 0.829 | -0.034 | 0.021 | 1.12E-01 | 1.17E-04 | 39 |
| 12 | 117360617 | rs7133066 | T | G | 0.850 | 0.033 | 0.006 | 4.21E-09 | 0.865 | -0.006 | 0.023 | 7.96E-01 | 9.18E-05 | 30 |
| 2 | 42970161 | rs10175423 | T | C | 0.297 | -0.025 | 0.004 | 4.25E-09 | 0.290 | -0.010 | 0.018 | 5.77E-01 | 1.19E-04 | 39 |
| 2 | 10368606 | rs7587651 | T | C | 0.373 | -0.024 | 0.004 | 4.26E-09 | 0.372 | 0.001 | 0.016 | 9.75E-01 | 1.09E-04 | 36 |
| 5 | 35030311 | rs10521021 | T | G | 0.658 | -0.024 | 0.004 | 4.77E-09 | 0.669 | 0.020 | 0.017 | 2.38E-01 | 1.09E-04 | 36 |
| 8 | 76591987 | rs1449543* | T | C | 0.456 | 0.022 | 0.004 | 5.06E-09 | 0.449 | -0.007 | 0.016 | 6.60E-01 | 9.18E-05 | 30 |
| 8 | 1523903 | rs7004265 | T | C | 0.477 | 0.023 | 0.004 | 5.12E-09 | 0.470 | -0.014 | 0.016 | 3.70E-01 | 1.00E-04 | 33 |
| 7 | 93215658 | rs149226155 | A | G | 0.347 | -0.024 | 0.004 | 5.67E-09 | 0.361 | -0.018 | 0.016 | 2.80E-01 | 1.09E-04 | 36 |
| 12 | 75978358 | rs1148006 | A | G | 0.244 | -0.026 | 0.004 | 5.81E-09 | 0.231 | -0.007 | 0.019 | 7.02E-01 | 1.28E-04 | 42 |
| 16 | 52283158 | rs143461173 | A | G | 0.806 | 0.029 | 0.005 | 6.10E-09 | 0.810 | 0.009 | 0.020 | 6.50E-01 | 1.02E-04 | 34 |
| 12 | 97506357 | rs7979001 | A | G | 0.508 | 0.022 | 0.004 | 6.13E-09 | 0.525 | 0.027 | 0.016 | 8.87E-02 | 9.18E-05 | 30 |
| 3 | 68595634 | rs7431217 | T | C | 0.412 | 0.023 | 0.004 | 6.19E-09 | 0.412 | -0.004 | 0.016 | 8.04E-01 | 1.00E-04 | 33 |
| 15 | 40608820 | rs34513772 | T | C | 0.670 | 0.024 | 0.004 | 6.20E-09 | 0.670 | 0.007 | 0.017 | 6.61E-01 | 1.09E-04 | 36 |
| 18 | 31765736 | rs8087304 | A | T | 0.482 | 0.022 | 0.004 | 6.54E-09 | 0.483 | -0.023 | 0.016 | 1.46E-01 | 9.18E-05 | 30 |
| 2 | 24106445 | rs150821390 | T | C | 0.028 | 0.072 | 0.012 | 6.59E-09 | 0.029 | -0.001 | 0.049 | 9.81E-01 | 1.09E-04 | 36 |
| 11 | 28899164 | rs6484408 | A | G | 0.287 | -0.025 | 0.004 | 6.63E-09 | 0.266 | 0.047 | 0.018 | 8.35E-03 | 1.19E-04 | 39 |
| 14 | 30514335 | rs10136330* | T | C | 0.043 | -0.058 | 0.010 | 6.65E-09 | 0.040 | 0.008 | 0.041 | 8.48E-01 | 1.02E-04 | 34 |
| 3 | 44883523 | rs77955256 | A | T | 0.106 | -0.036 | 0.006 | 7.10E-09 | 0.107 | 0.035 | 0.025 | 1.62E-01 | 1.09E-04 | 36 |
| 17 | 1942577 | rs112752732 | C | G | 0.046 | 0.057 | 0.010 | 7.26E-09 | 0.048 | 0.023 | 0.038 | 5.45E-01 | 9.86E-05 | 32 |
| 19 | 58973929 | rs2889128 | A | C | 0.456 | 0.022 | 0.004 | 7.57E-09 | 0.453 | 0.025 | 0.016 | 1.09E-01 | 9.18E-05 | 30 |
| 1 | 165426193 | rs7542538 | T | C | 0.813 | 0.029 | 0.005 | 7.63E-09 | 0.822 | -0.004 | 0.021 | 8.38E-01 | 1.02E-04 | 34 |
| 10 | 74071178 | rs4746113 | A | G | 0.309 | -0.024 | 0.004 | 8.79E-09 | 0.297 | 0.031 | 0.017 | 7.48E-02 | 1.09E-04 | 36 |
| 14 | 99709702 | rs941520 | A | C | 0.494 | -0.022 | 0.004 | 8.99E-09 | 0.493 | 0.012 | 0.016 | 4.50E-01 | 9.18E-05 | 30 |
| 14 | 101353211 | rs6575806 | A | C | 0.739 | -0.033 | 0.006 | 9.05E-09 | 0.720 | -0.003 | 0.019 | 8.55E-01 | 9.18E-05 | 30 |
| 17 | 46227846 | rs11079810* | T | C | 0.105 | 0.036 | 0.006 | 9.62E-09 | 0.103 | -0.011 | 0.026 | 6.66E-01 | 1.09E-04 | 36 |
| 4 | 104247262 | rs55784701 | T | C | 0.225 | 0.026 | 0.005 | 1.08E-08 | 0.221 | -0.048 | 0.019 | 1.23E-02 | 8.21E-05 | 27 |
| 8 | 53163528 | rs4487799 | A | T | 0.303 | 0.024 | 0.004 | 1.09E-08 | 0.294 | 0.008 | 0.017 | 6.53E-01 | 1.09E-04 | 36 |
| 13 | 112082513 | rs11619721 | T | G | 0.084 | -0.041 | 0.007 | 1.33E-08 | 0.093 | -0.074 | 0.028 | 7.57E-03 | 1.04E-04 | 34 |
| 13 | 42646769 | rs73187215 | A | G | 0.903 | -0.038 | 0.007 | 1.34E-08 | 0.910 | -0.014 | 0.028 | 6.26E-01 | 8.95E-05 | 29 |
| 9 | 136905474 | rs467379 | T | C | 0.307 | 0.024 | 0.004 | 1.35E-08 | 0.289 | 0.034 | 0.017 | 4.87E-02 | 1.09E-04 | 36 |
| 3 | 107700952 | rs709488 | A | C | 0.534 | -0.022 | 0.004 | 1.38E-08 | 0.526 | -0.026 | 0.016 | 9.58E-02 | 9.18E-05 | 30 |
| 2 | 32626815 | rs72787520 | G | C | 0.034 | 0.065 | 0.011 | 1.38E-08 | 0.039 | 0.041 | 0.040 | 3.14E-01 | 1.06E-04 | 35 |
| 7 | 50576648 | rs1470750* | C | G | 0.592 | -0.022 | 0.004 | 1.44E-08 | 0.588 | 0.026 | 0.016 | 1.13E-01 | 9.18E-05 | 30 |
| 7 | 130409054 | rs17563472 | T | C | 0.965 | -0.061 | 0.011 | 1.52E-08 | 0.962 | 0.045 | 0.042 | 2.81E-01 | 9.34E-05 | 31 |
| 1 | 154141908 | rs4845364 | A | G | 0.492 | 0.022 | 0.004 | 1.62E-08 | 0.494 | -0.026 | 0.016 | 9.83E-02 | 9.18E-05 | 30 |
| 6 | 170652191 | rs910425 | A | G | 0.448 | -0.022 | 0.004 | 1.63E-08 | 0.453 | 0.002 | 0.016 | 8.82E-01 | 9.18E-05 | 30 |
| 3 | 1906245 | rs73820560 | A | C | 0.856 | -0.032 | 0.006 | 1.68E-08 | 0.857 | 0.020 | 0.023 | 3.92E-01 | 8.64E-05 | 28 |
| 3 | 156532953 | rs11711674 | T | C | 0.571 | 0.022 | 0.004 | 1.77E-08 | 0.578 | 0.011 | 0.016 | 4.79E-01 | 9.18E-05 | 30 |
| 2 | 206956138 | rs184033703 | A | G | 0.058 | -0.048 | 0.009 | 1.85E-08 | 0.057 | -0.024 | 0.034 | 4.85E-01 | 8.64E-05 | 28 |
| 5 | 77048448 | rs13173441 | T | C | 0.879 | 0.033 | 0.006 | 1.86E-08 | 0.886 | -0.028 | 0.025 | 2.61E-01 | 9.18E-05 | 30 |
| 2 | 60175475 | rs1025128* | C | G | 0.568 | -0.022 | 0.004 | 1.88E-08 | 0.564 | 0.023 | 0.016 | 1.52E-01 | 9.18E-05 | 30 |
| 20 | 31054702 | rs1737894 | C | G | 0.600 | 0.022 | 0.004 | 1.91E-08 | 0.613 | -0.007 | 0.016 | 6.54E-01 | 9.18E-05 | 30 |
| 9 | 81679875 | rs4877387 | T | C | 0.282 | 0.024 | 0.004 | 2.20E-08 | 0.284 | -0.033 | 0.018 | 6.31E-02 | 1.09E-04 | 36 |
| 1 | 38940215 | rs4970598 | T | C | 0.959 | 0.062 | 0.011 | 2.34E-08 | 0.964 | 0.040 | 0.044 | 3.58E-01 | 9.65E-05 | 32 |
| 20 | 43529461 | rs2425674 | C | G | 0.439 | -0.022 | 0.004 | 2.35E-08 | 0.438 | -0.033 | 0.016 | 3.56E-02 | 9.18E-05 | 30 |
| 3 | 84462073 | rs7426534 | A | G | 0.709 | -0.024 | 0.004 | 2.39E-08 | 0.703 | -0.019 | 0.017 | 2.71E-01 | 1.09E-04 | 36 |
| 7 | 114296102 | rs1456031 | T | C | 0.465 | 0.022 | 0.004 | 2.44E-08 | 0.446 | -0.021 | 0.016 | 1.83E-01 | 9.18E-05 | 30 |
| 6 | 146687748 | rs117530880 | T | G | 0.970 | -0.066 | 0.012 | 2.58E-08 | 0.968 | 0.027 | 0.045 | 5.46E-01 | 9.18E-05 | 30 |
| 6 | 124253495 | rs235696 | C | G | 0.339 | 0.023 | 0.004 | 2.66E-08 | 0.346 | 0.003 | 0.017 | 8.47E-01 | 1.00E-04 | 33 |
| 10 | 71380093 | rs7072571 | A | G | 0.784 | 0.031 | 0.006 | 2.69E-08 | 0.788 | -0.001 | 0.019 | 9.61E-01 | 8.10E-05 | 27 |
| 11 | 22791324 | rs1032682 | T | C | 0.407 | 0.021 | 0.004 | 2.69E-08 | 0.401 | -0.003 | 0.016 | 8.33E-01 | 8.37E-05 | 28 |
| 6 | 14918298 | rs446745 | T | C | 0.241 | -0.026 | 0.005 | 2.81E-08 | 0.244 | 0.021 | 0.018 | 2.43E-01 | 8.21E-05 | 27 |
| 5 | 59140876 | rs256350 | T | C | 0.726 | -0.024 | 0.004 | 2.84E-08 | 0.730 | 0.003 | 0.018 | 8.80E-01 | 1.09E-04 | 36 |
| 10 | 112759731 | rs10885077 | T | G | 0.255 | 0.024 | 0.004 | 2.95E-08 | 0.261 | -0.017 | 0.018 | 3.33E-01 | 1.09E-04 | 36 |
| 1 | 27212209 | rs141847393 | T | C | 0.918 | 0.040 | 0.007 | 2.96E-08 | 0.920 | -0.051 | 0.029 | 7.81E-02 | 9.91E-05 | 33 |
| 2 | 213403972 | rs6735626* | A | G | 0.437 | 0.022 | 0.004 | 2.96E-08 | 0.436 | 0.000 | 0.016 | 9.86E-01 | 9.18E-05 | 30 |
| 1 | 54728858 | rs643428* | T | C | 0.593 | -0.022 | 0.004 | 3.21E-08 | 0.606 | -0.007 | 0.016 | 6.76E-01 | 9.18E-05 | 30 |
| 17 | 2017993 | rs142643995 | T | C | 0.030 | 0.065 | 0.012 | 3.25E-08 | 0.032 | -0.011 | 0.046 | 8.11E-01 | 8.91E-05 | 29 |
| 10 | 10251910 | rs1885740 | A | G | 0.281 | -0.026 | 0.005 | 3.35E-08 | 0.285 | 0.026 | 0.018 | 1.50E-01 | 8.21E-05 | 27 |
| 12 | 121300988 | rs660549 | T | C | 0.567 | -0.021 | 0.004 | 3.36E-08 | 0.564 | 0.015 | 0.016 | 3.40E-01 | 8.37E-05 | 28 |
| 11 | 45433845 | rs970179 | A | G | 0.472 | 0.021 | 0.004 | 3.53E-08 | 0.476 | 0.014 | 0.016 | 3.69E-01 | 8.37E-05 | 28 |
| 19 | 49209325 | rs2548458 | T | C | 0.506 | 0.021 | 0.004 | 3.53E-08 | 0.539 | 0.002 | 0.016 | 8.98E-01 | 8.37E-05 | 28 |
| 7 | 129663496 | rs11556924 | T | C | 0.370 | 0.023 | 0.004 | 3.66E-08 | 0.386 | 0.034 | 0.016 | 3.78E-02 | 1.00E-04 | 33 |
| 7 | 27763590 | rs10268051 | A | C | 0.777 | 0.025 | 0.005 | 3.88E-08 | 0.776 | 0.032 | 0.019 | 9.04E-02 | 7.59E-05 | 25 |
| 6 | 84286477 | rs11756746 | A | G | 0.243 | 0.025 | 0.005 | 3.95E-08 | 0.245 | -0.032 | 0.018 | 8.21E-02 | 7.59E-05 | 25 |
| 2 | 172701157 | rs2271758* | T | G | 0.411 | -0.021 | 0.004 | 3.96E-08 | 0.411 | -0.019 | 0.016 | 2.31E-01 | 8.37E-05 | 28 |
| 18 | 45921214 | rs7239114* | A | G | 0.540 | -0.022 | 0.004 | 4.02E-08 | 0.545 | 0.020 | 0.016 | 2.10E-01 | 9.18E-05 | 30 |
| 13 | 49475780 | rs9568123 | A | G | 0.848 | -0.029 | 0.005 | 4.06E-08 | 0.844 | 0.040 | 0.022 | 6.96E-02 | 1.02E-04 | 34 |
| 1 | 154631123 | rs9427116 | T | C | 0.488 | 0.021 | 0.004 | 4.24E-08 | 0.482 | 0.027 | 0.016 | 8.13E-02 | 8.37E-05 | 28 |
| 14 | 101367407 | rs79084266 | C | G | 0.881 | -0.040 | 0.007 | 4.27E-08 | 0.877 | 0.056 | 0.025 | 2.63E-02 | 9.91E-05 | 33 |
| 2 | 137613322 | rs2558101 | A | G | 0.720 | -0.024 | 0.004 | 4.28E-08 | 0.693 | -0.037 | 0.017 | 3.10E-02 | 1.09E-04 | 36 |
| 1 | 179732142 | rs61828391 | A | G | 0.126 | -0.032 | 0.006 | 4.31E-08 | 0.128 | 0.025 | 0.024 | 2.88E-01 | 8.64E-05 | 28 |
| 16 | 72569236 | rs4448948* | A | T | 0.929 | -0.041 | 0.008 | 4.33E-08 | 0.924 | 0.049 | 0.030 | 1.01E-01 | 7.97E-05 | 26 |
| 7 | 78139581 | rs1030015* | T | G | 0.522 | -0.021 | 0.004 | 4.45E-08 | 0.516 | 0.033 | 0.016 | 3.48E-02 | 8.37E-05 | 28 |
| 6 | 22562485 | rs1539310 | A | G | 0.757 | 0.024 | 0.005 | 4.59E-08 | 0.756 | -0.004 | 0.018 | 8.15E-01 | 7.00E-05 | 23 |
| 10 | 121154531 | rs73435048 | A | G | 0.067 | -0.044 | 0.008 | 4.94E-08 | 0.064 | 0.009 | 0.033 | 7.82E-01 | 9.18E-05 | 30 |
| **AAM (12)** | | | | | | | | | | | | | | |
| 6 | 104931079 | rs7759938 | C | T | 0.320 | 0.120 | 0.005 | 8.00E-110 | 0.320 | -0.010 | 0.017 | 5.52E-01 | 3.02E-03 | 553 |
| 9 | 106157939 | rs10453225 | G | T | 0.680 | 0.090 | 0.005 | 6.00E-66 | 0.685 | 0.020 | 0.017 | 2.39E-01 | 1.70E-03 | 311 |
| 6 | 104900669 | rs2153127 | T | C | 0.520 | 0.080 | 0.005 | 6.00E-59 | 0.525 | 0.010 | 0.016 | 5.26E-01 | 1.35E-03 | 246 |
| 9 | 106298549 | rs10739221* | C | T | 0.770 | 0.080 | 0.006 | 4.00E-41 | 0.780 | 0.023 | 0.019 | 2.37E-01 | 9.35E-04 | 171 |
| 2 | 56360614 | rs6747380 | A | G | 0.170 | 0.070 | 0.007 | 6.00E-28 | 0.166 | 0.034 | 0.021 | 1.04E-01 | 5.26E-04 | 96 |
| 3 | 117843589 | rs11715566 | T | C | 0.500 | 0.050 | 0.005 | 2.00E-27 | 0.492 | 0.017 | 0.016 | 2.95E-01 | 5.26E-04 | 96 |
| 11 | 122974367 | rs1461503 | C | A | 0.570 | 0.050 | 0.005 | 3.00E-26 | 0.552 | -0.009 | 0.016 | 5.62E-01 | 5.26E-04 | 96 |
| 1 | 165425645 | rs466639 | C | T | 0.870 | 0.080 | 0.007 | 2.00E-24 | 0.874 | -0.015 | 0.024 | 5.34E-01 | 6.87E-04 | 125 |
| 17 | 51536424 | rs9635759 | A | G | 0.320 | 0.050 | 0.005 | 2.00E-24 | 0.304 | -0.012 | 0.017 | 4.72E-01 | 5.26E-04 | 96 |
| 7 | 41430495 | rs1079866 | G | C | 0.150 | 0.070 | 0.007 | 9.00E-24 | 0.133 | 0.039 | 0.023 | 8.89E-02 | 5.26E-04 | 96 |
| 9 | 111517632 | rs10980921 | C | T | 0.090 | 0.090 | 0.009 | 2.00E-23 | 0.082 | -0.003 | 0.029 | 9.07E-01 | 5.26E-04 | 96 |
| 16 | 69554669 | rs1364063* | C | T | 0.430 | 0.050 | 0.005 | 6.00E-21 | 0.409 | -0.017 | 0.016 | 3.04E-01 | 5.26E-04 | 96 |
| 5 | 134564823 | rs13179411 | T | G | 0.170 | 0.060 | 0.007 | 3.00E-20 | 0.151 | 0.014 | 0.022 | 5.19E-01 | 3.87E-04 | 71 |
| 11 | 13293892 | rs11022756* | A | C | 0.290 | 0.050 | 0.006 | 7.00E-20 | 0.291 | 0.015 | 0.017 | 3.83E-01 | 3.65E-04 | 67 |
| 18 | 47225754 | rs2137289 | G | A | 0.410 | -0.050 | 0.005 | 8.00E-20 | 0.413 | -0.011 | 0.016 | 4.81E-01 | 5.26E-04 | 96 |
| 2 | 198773966 | rs1400974 | A | G | 0.640 | 0.050 | 0.005 | 8.00E-20 | 0.643 | -0.004 | 0.017 | 8.15E-01 | 5.26E-04 | 96 |
| 2 | 614168 | rs2947411* | A | G | 0.170 | 0.060 | 0.007 | 2.00E-19 | 0.173 | -0.056 | 0.021 | 7.21E-03 | 3.87E-04 | 71 |
| 2 | 199410486 | rs17266097 | T | C | 0.420 | 0.040 | 0.005 | 3.00E-18 | 0.399 | -0.019 | 0.016 | 2.50E-01 | 3.37E-04 | 61 |
| 16 | 53782363 | rs8050136* | C | A | 0.600 | 0.040 | 0.005 | 2.00E-17 | 0.607 | -0.037 | 0.016 | 2.10E-02 | 3.37E-04 | 61 |
| 13 | 111531001 | rs9560113* | G | A | 0.280 | 0.050 | 0.006 | 2.00E-17 | 0.287 | -0.013 | 0.017 | 4.41E-01 | 3.65E-04 | 67 |
| 8 | 77181601 | rs7821178 | A | C | 0.350 | -0.040 | 0.005 | 7.00E-17 | 0.335 | 0.014 | 0.017 | 3.97E-01 | 3.37E-04 | 61 |
| 3 | 185917897 | rs16860328 | G | A | 0.420 | 0.040 | 0.005 | 1.00E-16 | 0.432 | -0.045 | 0.016 | 4.83E-03 | 3.37E-04 | 61 |
| 3 | 49473498 | rs7647973 | A | G | 0.260 | 0.050 | 0.006 | 1.00E-16 | 0.256 | 0.014 | 0.018 | 4.23E-01 | 3.65E-04 | 67 |
| 14 | 60453807 | rs1254337 | T | A | 0.310 | 0.040 | 0.005 | 2.00E-16 | 0.304 | 0.017 | 0.017 | 3.11E-01 | 3.37E-04 | 61 |
| 1 | 74541036 | rs7514705* | C | T | 0.560 | 0.040 | 0.005 | 2.00E-16 | 0.563 | -0.004 | 0.016 | 7.80E-01 | 3.37E-04 | 61 |
| 3 | 86867732 | rs7642134 | G | A | 0.610 | 0.040 | 0.005 | 3.00E-16 | 0.617 | -0.028 | 0.016 | 8.25E-02 | 3.37E-04 | 61 |
| 6 | 99668216 | rs9321659 | A | G | 0.130 | 0.060 | 0.008 | 3.00E-16 | 0.134 | -0.003 | 0.023 | 9.14E-01 | 2.96E-04 | 54 |
| 16 | 14301575 | rs246185 | C | T | 0.330 | 0.040 | 0.006 | 7.00E-16 | 0.321 | 0.045 | 0.017 | 8.01E-03 | 2.44E-04 | 44 |
| 1 | 177920345 | rs543874* | A | G | 0.800 | 0.050 | 0.006 | 1.00E-15 | 0.791 | -0.041 | 0.019 | 3.46E-02 | 3.65E-04 | 67 |
| 14 | 10041606 | rs10144321 | A | G | 0.750 | 0.040 | 0.006 | 9.00E-15 | 0.770 | -0.025 | 0.019 | 1.87E-01 | 2.34E-04 | 43 |
| 11 | 101565990 | rs10895140 | G | A | 0.660 | 0.040 | 0.005 | 7.00E-14 | 0.670 | 0.013 | 0.017 | 4.24E-01 | 3.37E-04 | 61 |
| 5 | 138389314 | rs17171818 | C | T | 0.770 | 0.040 | 0.006 | 9.00E-14 | 0.798 | -0.026 | 0.020 | 1.94E-01 | 2.34E-04 | 43 |
| 6 | 99760562 | rs4840086 | A | G | 0.580 | 0.040 | 0.005 | 9.00E-14 | 0.575 | 0.001 | 0.016 | 9.55E-01 | 3.37E-04 | 61 |
| 8 | 4702559 | rs7828501 | G | A | 0.450 | 0.040 | 0.005 | 1.00E-13 | 0.451 | -0.021 | 0.016 | 1.82E-01 | 3.37E-04 | 61 |
| 20 | 17141948 | rs852069* | G | A | 0.640 | 0.040 | 0.005 | 1.00E-13 | 0.642 | -0.023 | 0.016 | 1.69E-01 | 3.37E-04 | 61 |
| 15 | 60489314 | rs3743266 | T | C | 0.680 | 0.040 | 0.005 | 2.00E-13 | 0.668 | 0.004 | 0.017 | 8.29E-01 | 3.37E-04 | 61 |
| 15 | 67667126 | rs8032675* | T | C | 0.400 | 0.040 | 0.005 | 2.00E-13 | 0.397 | -0.012 | 0.016 | 4.50E-01 | 3.37E-04 | 61 |
| 19 | 9885191 | rs889122 | G | T | 0.720 | 0.040 | 0.006 | 2.00E-13 | 0.721 | -0.012 | 0.018 | 4.77E-01 | 2.34E-04 | 43 |
| 4 | 45180510 | rs10938397* | A | G | 0.570 | 0.040 | 0.005 | 4.00E-13 | 0.567 | -0.013 | 0.016 | 4.11E-01 | 3.37E-04 | 61 |
| 1 | 72285502 | rs3101336* | T | C | 0.400 | 0.040 | 0.005 | 5.00E-13 | 0.397 | -0.016 | 0.016 | 3.32E-01 | 3.37E-04 | 61 |
| 6 | 126460288 | rs4895808 | C | T | 0.540 | 0.030 | 0.005 | 5.00E-13 | 0.546 | 0.016 | 0.016 | 3.11E-01 | 1.90E-04 | 35 |
| 4 | 103719946 | rs3733631 | C | G | 0.150 | 0.050 | 0.007 | 5.00E-13 | 0.152 | -0.026 | 0.022 | 2.34E-01 | 2.69E-04 | 49 |
| 11 | 46129068 | rs4756059 | T | C | 0.920 | 0.070 | 0.010 | 5.00E-13 | 0.928 | -0.028 | 0.030 | 3.63E-01 | 2.58E-04 | 47 |
| 2 | 105248369 | rs6758290 | T | C | 0.500 | 0.040 | 0.005 | 7.00E-13 | 0.492 | -0.028 | 0.016 | 7.36E-02 | 3.37E-04 | 61 |
| 6 | 41923244 | rs2479724 | T | C | 0.450 | 0.030 | 0.005 | 1.00E-12 | 0.450 | -0.006 | 0.016 | 7.17E-01 | 1.90E-04 | 35 |
| 6 | 54891510 | rs988913 | C | T | 0.660 | 0.040 | 0.005 | 1.00E-12 | 0.650 | -0.042 | 0.017 | 1.14E-02 | 3.37E-04 | 61 |
| 12 | 49853685 | rs7138803* | G | A | 0.620 | 0.040 | 0.005 | 2.00E-12 | 0.629 | -0.034 | 0.016 | 3.49E-02 | 3.37E-04 | 61 |
| 10 | 1689677 | rs1874984 | C | G | 0.470 | 0.040 | 0.005 | 2.00E-12 | 0.493 | 0.004 | 0.016 | 8.18E-01 | 3.51E-04 | 64 |
| 11 | 78337478 | rs2063730 | C | A | 0.180 | 0.050 | 0.007 | 2.00E-12 | 0.196 | -0.001 | 0.020 | 9.65E-01 | 2.69E-04 | 49 |
| 3 | 24669522 | rs6770162 | A | G | 0.510 | 0.040 | 0.005 | 2.00E-12 | 0.500 | 0.020 | 0.016 | 2.17E-01 | 3.37E-04 | 61 |
| 15 | 88499236 | rs12915845 | C | T | 0.580 | 0.030 | 0.005 | 3.00E-12 | 0.576 | -0.024 | 0.016 | 1.31E-01 | 1.90E-04 | 35 |
| 11 | 8618447 | rs4929947* | G | C | 0.360 | 0.040 | 0.005 | 3.00E-12 | 0.354 | -0.010 | 0.016 | 5.37E-01 | 3.37E-04 | 61 |
| 3 | 184328011 | rs939317 | G | A | 0.740 | 0.040 | 0.006 | 3.00E-12 | 0.744 | 0.020 | 0.018 | 2.67E-01 | 2.34E-04 | 43 |
| 8 | 139639536 | rs1469039 | A | G | 0.190 | 0.050 | 0.007 | 4.00E-12 | 0.178 | 0.002 | 0.021 | 9.41E-01 | 2.69E-04 | 49 |
| 7 | 74687575 | rs6964833* | T | C | 0.750 | 0.040 | 0.006 | 5.00E-12 | 0.740 | -0.020 | 0.018 | 2.70E-01 | 2.34E-04 | 43 |
| 6 | 100312294 | rs13196561 | C | A | 0.780 | 0.040 | 0.006 | 8.00E-12 | 0.783 | 0.009 | 0.019 | 6.46E-01 | 2.34E-04 | 43 |
| 3 | 50055776 | rs6762477* | G | A | 0.440 | 0.040 | 0.006 | 8.00E-12 | 0.447 | 0.060 | 0.016 | 1.46E-04 | 2.34E-04 | 43 |
| 6 | 56915943 | rs9475752 | C | T | 0.810 | 0.040 | 0.006 | 8.00E-12 | 0.804 | -0.026 | 0.020 | 1.94E-01 | 2.34E-04 | 43 |
| 19 | 18707093 | rs10423674* | A | C | 0.340 | 0.040 | 0.005 | 9.00E-12 | 0.334 | -0.005 | 0.017 | 7.70E-01 | 3.37E-04 | 61 |
| 16 | 70011845 | rs929843* | A | C | 0.230 | 0.040 | 0.006 | 1.00E-11 | 0.217 | -0.031 | 0.019 | 1.07E-01 | 2.34E-04 | 43 |
| 5 | 167943258 | rs9647570 | G | T | 0.140 | 0.050 | 0.007 | 1.00E-11 | 0.141 | -0.015 | 0.023 | 5.23E-01 | 2.69E-04 | 49 |
| 9 | 109047015 | rs11792861 | A | C | 0.700 | 0.040 | 0.005 | 2.00E-11 | 0.707 | 0.004 | 0.017 | 8.35E-01 | 3.37E-04 | 61 |
| 13 | 39665648 | rs6563739 | G | T | 0.340 | 0.030 | 0.005 | 2.00E-11 | 0.337 | -0.004 | 0.017 | 8.04E-01 | 1.90E-04 | 35 |
| 3 | 157080986 | rs900400 | T | C | 0.610 | 0.030 | 0.005 | 2.00E-11 | 0.608 | 0.013 | 0.017 | 4.37E-01 | 1.90E-04 | 35 |
| 4 | 28750432 | rs1038903 | T | C | 0.730 | 0.040 | 0.006 | 2.00E-11 | 0.731 | -0.017 | 0.018 | 3.46E-01 | 2.34E-04 | 43 |
| 11 | 27678578 | rs7103411* | C | T | 0.210 | 0.040 | 0.006 | 3.00E-11 | 0.208 | -0.027 | 0.020 | 1.70E-01 | 2.34E-04 | 43 |
| 3 | 132896620 | rs2600959 | A | G | 0.340 | 0.040 | 0.005 | 4.00E-11 | 0.350 | 0.013 | 0.017 | 4.27E-01 | 3.37E-04 | 61 |
| 7 | 122520688 | rs11767400 | A | C | 0.300 | 0.040 | 0.006 | 4.00E-11 | 0.305 | 0.002 | 0.017 | 8.94E-01 | 2.34E-04 | 43 |
| 17 | 55153361 | rs244293 | G | A | 0.600 | 0.030 | 0.005 | 4.00E-11 | 0.626 | 0.006 | 0.016 | 7.20E-01 | 1.90E-04 | 35 |
| 15 | 23906947 | rs12148769 | G | A | 0.900 | 0.050 | 0.008 | 5.00E-11 | 0.895 | -0.004 | 0.026 | 8.89E-01 | 2.06E-04 | 38 |
| 18 | 3817134 | rs12607903 | C | T | 0.300 | 0.040 | 0.005 | 5.00E-11 | 0.278 | 0.005 | 0.018 | 7.87E-01 | 3.37E-04 | 61 |
| 2 | 198779314 | rs17233066 | C | T | 0.930 | 0.090 | 0.014 | 6.00E-11 | 0.934 | 0.025 | 0.032 | 4.33E-01 | 2.34E-04 | 43 |
| 21 | 39232503 | rs2836950* | C | G | 0.640 | 0.030 | 0.005 | 6.00E-11 | 0.644 | -0.029 | 0.016 | 7.71E-02 | 1.97E-04 | 36 |
| 9 | 7174673 | rs913588 | G | A | 0.490 | 0.030 | 0.005 | 6.00E-11 | 0.481 | -0.002 | 0.016 | 8.84E-01 | 1.97E-04 | 36 |
| 11 | 115181915 | rs11215400* | C | A | 0.270 | 0.040 | 0.006 | 7.00E-11 | 0.259 | 0.014 | 0.018 | 4.21E-01 | 2.34E-04 | 43 |
| 3 | 128176383 | rs2687729 | G | A | 0.270 | 0.040 | 0.006 | 1.00E-10 | 0.266 | -0.019 | 0.018 | 2.89E-01 | 2.34E-04 | 43 |
| 4 | 94286537 | rs13135934 | C | G | 0.400 | 0.030 | 0.005 | 1.10E-10 | 0.401 | -0.024 | 0.016 | 1.40E-01 | 1.97E-04 | 36 |
| 2 | 156270452 | rs4369815 | T | G | 0.930 | 0.060 | 0.010 | 2.00E-10 | 0.935 | -0.008 | 0.032 | 8.04E-01 | 1.90E-04 | 35 |
| 10 | 119949417 | rs12571664 | T | C | 0.790 | 0.040 | 0.006 | 3.00E-10 | 0.815 | -0.017 | 0.020 | 4.04E-01 | 2.34E-04 | 43 |
| 13 | 74061451 | rs1324913 | G | T | 0.650 | 0.030 | 0.005 | 3.00E-10 | 0.663 | 0.017 | 0.017 | 2.99E-01 | 1.90E-04 | 35 |
| 5 | 111524099 | rs251130 | G | A | 0.730 | 0.040 | 0.006 | 3.00E-10 | 0.726 | 0.028 | 0.018 | 1.20E-01 | 2.34E-04 | 43 |
| 6 | 29954963 | rs16896742 | G | A | 0.380 | 0.040 | 0.006 | 3.00E-10 | 0.360 | -0.003 | 0.016 | 8.76E-01 | 2.34E-04 | 43 |
| 2 | 141470940 | rs12472911 | C | T | 0.200 | 0.040 | 0.006 | 7.00E-10 | 0.227 | -0.016 | 0.019 | 4.07E-01 | 2.34E-04 | 43 |
| 6 | 151482619 | rs6933660 | C | A | 0.690 | 0.030 | 0.005 | 1.00E-09 | 0.684 | 0.020 | 0.017 | 2.37E-01 | 1.90E-04 | 35 |
| 3 | 137271149 | rs13067731 | T | C | 0.160 | 0.040 | 0.007 | 1.00E-09 | 0.164 | 0.028 | 0.021 | 1.85E-01 | 1.72E-04 | 31 |
| 1 | 97909892 | rs11165924 | A | G | 0.690 | 0.030 | 0.006 | 2.00E-09 | 0.667 | 0.025 | 0.017 | 1.31E-01 | 1.32E-04 | 24 |
| 16 | 29906713 | rs1129700* | T | C | 0.440 | 0.030 | 0.005 | 2.00E-09 | 0.433 | -0.022 | 0.016 | 1.63E-01 | 1.90E-04 | 35 |
| 1 | 43655886 | rs2274465 | C | G | 0.660 | 0.030 | 0.005 | 2.00E-09 | 0.656 | 0.031 | 0.017 | 6.16E-02 | 1.90E-04 | 35 |
| 8 | 3909688 | rs2688325 | T | C | 0.290 | 0.030 | 0.006 | 2.00E-09 | 0.299 | 0.009 | 0.017 | 5.91E-01 | 1.32E-04 | 24 |
| 5 | 169322733 | rs6555855 | G | A | 0.230 | 0.040 | 0.006 | 2.00E-09 | 0.208 | 0.016 | 0.019 | 4.04E-01 | 2.34E-04 | 43 |
| 6 | 128069835 | rs6938574 | T | C | 0.160 | 0.040 | 0.007 | 2.00E-09 | 0.169 | 0.013 | 0.021 | 5.49E-01 | 1.72E-04 | 31 |
| 9 | 84100651 | rs7853970 | T | C | 0.470 | 0.030 | 0.005 | 2.00E-09 | 0.463 | -0.021 | 0.016 | 1.88E-01 | 1.90E-04 | 35 |
| 2 | 155895947 | rs17236969 | T | C | 0.140 | 0.050 | 0.008 | 3.00E-09 | 0.159 | 0.031 | 0.022 | 1.51E-01 | 2.06E-04 | 38 |
| 5 | 43116728 | rs1532331* | G | T | 0.320 | 0.030 | 0.005 | 4.00E-09 | 0.305 | -0.006 | 0.017 | 7.38E-01 | 1.90E-04 | 35 |
| 9 | 6942940 | rs7037266* | A | C | 0.370 | 0.030 | 0.005 | 5.00E-09 | 0.363 | 0.003 | 0.016 | 8.57E-01 | 1.90E-04 | 35 |
| 14 | 100716133 | rs7141210 | T | C | 0.340 | 0.030 | 0.005 | 6.00E-09 | 0.322 | 0.019 | 0.017 | 2.62E-01 | 1.90E-04 | 35 |
| 6 | 76458369 | rs9447700 | C | T | 0.690 | 0.030 | 0.005 | 6.00E-09 | 0.695 | -0.034 | 0.017 | 4.82E-02 | 1.90E-04 | 35 |
| 8 | 52856653 | rs16918254 | A | G | 0.920 | 0.050 | 0.009 | 1.00E-08 | 0.929 | -0.008 | 0.031 | 7.90E-01 | 1.62E-04 | 30 |
| 8 | 4976268 | rs7463166 | A | G | 0.630 | 0.030 | 0.005 | 1.00E-08 | 0.624 | -0.011 | 0.016 | 4.89E-01 | 1.90E-04 | 35 |
| 9 | 111288077 | rs10980854 | A | G | 0.060 | 0.060 | 0.011 | 1.00E-08 | 0.050 | 0.004 | 0.036 | 9.16E-01 | 1.57E-04 | 29 |
| 16 | 19924067 | rs12446632* | A | G | 0.130 | 0.040 | 0.007 | 1.00E-08 | 0.143 | -0.053 | 0.023 | 2.01E-02 | 1.72E-04 | 31 |
| 8 | 143790241 | rs4875053 | G | C | 0.040 | 0.030 | 0.006 | 1.00E-08 | 0.426 | 0.010 | 0.016 | 5.45E-01 | 1.37E-04 | 25 |
| 19 | 7835676 | rs652260 | T | C | 0.540 | 0.030 | 0.005 | 1.00E-08 | 0.558 | 0.009 | 0.016 | 5.75E-01 | 1.90E-04 | 35 |
| 12 | 47486366 | rs7955374 | T | C | 0.130 | 0.040 | 0.008 | 1.00E-08 | 0.120 | -0.002 | 0.025 | 9.24E-01 | 1.32E-04 | 24 |
| 9 | 105995389 | rs10816359 | T | G | 0.860 | 0.040 | 0.008 | 2.00E-08 | 0.835 | -0.011 | 0.021 | 5.96E-01 | 1.32E-04 | 24 |
| 17 | 6131511 | rs7215990 | G | A | 0.760 | 0.040 | 0.006 | 2.00E-08 | 0.753 | -0.019 | 0.018 | 3.07E-01 | 2.34E-04 | 43 |
| 3 | 88192124 | rs9849248* | C | T | 0.150 | 0.040 | 0.007 | 2.00E-08 | 0.155 | 0.024 | 0.022 | 2.68E-01 | 1.72E-04 | 31 |
| 22 | 38849613 | rs13053505 | G | T | 0.800 | 0.040 | 0.007 | 3.00E-08 | 0.829 | 0.034 | 0.021 | 1.10E-01 | 1.72E-04 | 31 |
| 11 | 29102635 | rs16918636 | T | C | 0.790 | 0.030 | 0.006 | 3.00E-08 | 0.782 | -0.008 | 0.019 | 6.70E-01 | 1.32E-04 | 24 |
| 6 | 100686201 | rs239198* | T | C | 0.460 | 0.030 | 0.005 | 3.00E-08 | 0.467 | 0.011 | 0.016 | 4.82E-01 | 1.90E-04 | 35 |
| 2 | 59653910 | rs268067* | A | G | 0.800 | 0.040 | 0.006 | 3.00E-08 | 0.812 | -0.023 | 0.020 | 2.61E-01 | 2.34E-04 | 43 |
| 1 | 65350884 | rs10789181 | A | G | 0.390 | 0.030 | 0.005 | 4.00E-08 | 0.396 | -0.009 | 0.016 | 5.71E-01 | 1.90E-04 | 35 |
| 5 | 96510150 | rs17086188 | A | G | 0.940 | 0.070 | 0.013 | 4.00E-08 | 0.973 | -0.060 | 0.049 | 2.17E-01 | 1.65E-04 | 30 |
| 11 | 229977 | rs7104764 | G | A | 0.250 | 0.030 | 0.006 | 4.00E-08 | 0.248 | -0.012 | 0.018 | 5.08E-01 | 1.32E-04 | 24 |
| 10 | 125157645 | rs1915146 | G | A | 0.400 | 0.030 | 0.005 | 4.00E-08 | 0.420 | 0.031 | 0.016 | 5.67E-02 | 1.90E-04 | 35 |
| 14 | 65570077 | rs1958560 | A | G | 0.590 | 0.030 | 0.005 | 4.00E-08 | 0.605 | 0.014 | 0.016 | 3.98E-01 | 1.90E-04 | 35 |
| 22 | 49281720 | rs6009583 | C | T | 0.740 | 0.030 | 0.006 | 5.00E-08 | 0.732 | 0.004 | 0.018 | 8.15E-01 | 1.32E-04 | 24 |
| 1 | 102111465 | rs11578152 | G | A | 0.440 | 0.030 | 0.005 | 5.00E-08 | 0.451 | -0.024 | 0.016 | 1.27E-01 | 1.90E-04 | 35 |
| 1 | 199829211 | rs6427782 | A | G | 0.510 | 0.030 | 0.005 | 5.00E-08 | 0.513 | 0.031 | 0.016 | 4.84E-02 | 1.90E-04 | 35 |
| 5 | 154167849 | rs7701886* | A | G | 0.580 | 0.030 | 0.005 | 5.00E-08 | 0.545 | -0.022 | 0.016 | 1.78E-01 | 1.90E-04 | 35 |
| 9 | 10274080 | rs7865468 | A | G | 0.700 | 0.030 | 0.005 | 1.00E-07 | 0.705 | -0.001 | 0.018 | 9.49E-01 | 1.90E-04 | 35 |

* : snp associated with BMI

**Supplementary Table 3:** Detailed information for instrumental variables of age at natural menopause (ANM).

| **Chr** | **Position**  **(hg19)** | **rsID** | **Effect Allele** | **Other Allele** | **Exposure** | | | | **Outcome** | | | | **R^2^** | **F-statistic** |
| --- | --- | --- | --- | --- | --- | --- | --- | --- | --- | --- | --- | --- | --- | --- |
|  |  |  |  |  | **EAF** | **Beta** | **SE** | **p** | **EAF** | **Beta** | **SE** | **p** |  |  |
| 20 | 5896227 | rs16991615 | G | A | 0.930 | -0.880 | 0.040 | 1.60E-89 | 0.935 | 0.000 | 0.032 | 9.94E-01 | 6.93E-03 | 484 |
| 19 | 60525476 | rs11668344 | G | A | 0.360 | -0.410 | 0.020 | 5.50E-85 | 0.071 | -0.030 | 0.016 | 7.05E-02 | 6.02E-03 | 420 |
| 5 | 176311180 | rs365132 | G | T | 0.510 | -0.240 | 0.020 | 1.40E-33 | 0.518 | -0.012 | 0.016 | 4.39E-01 | 2.07E-03 | 144 |
| 4 | 84592646 | rs4693089 | A | G | 0.510 | -0.200 | 0.020 | 9.20E-23 | 0.507 | 0.007 | 0.016 | 6.40E-01 | 1.44E-03 | 100 |
| 8 | 38099744 | rs2720044 | A | C | 0.840 | -0.290 | 0.030 | 7.30E-22 | 0.842 | -0.018 | 0.022 | 4.08E-01 | 1.35E-03 | 93 |
| 1 | 39152972 | rs4246511 | C | T | 0.710 | -0.220 | 0.020 | 5.10E-21 | 0.123 | -0.027 | 0.018 | 1.23E-01 | 1.74E-03 | 121 |
| 15 | 87680130 | rs1054875 | T | A | 0.400 | -0.190 | 0.020 | 1.70E-19 | 0.427 | 0.013 | 0.016 | 4.27E-01 | 1.30E-03 | 90 |
| 12 | 55432336 | rs2277339 | G | T | 0.100 | -0.310 | 0.030 | 1.80E-19 | 0.213 | 0.032 | 0.026 | 2.13E-01 | 1.54E-03 | 107 |
| 6 | 11003246 | rs6899676 | A | G | 0.800 | -0.230 | 0.030 | 2.20E-19 | 0.796 | -0.019 | 0.020 | 3.25E-01 | 8.47E-04 | 59 |
| 2 | 171649264 | rs930036 | A | G | 0.380 | -0.190 | 0.020 | 3.10E-19 | 0.086 | 0.028 | 0.016 | 8.58E-02 | 1.30E-03 | 90 |
| 13 | 60011740 | rs4886238 | G | A | 0.660 | -0.180 | 0.020 | 2.50E-16 | 0.667 | 0.010 | 0.017 | 5.70E-01 | 1.17E-03 | 81 |
| 19 | 61012475 | rs12461110 | A | G | 0.350 | -0.170 | 0.020 | 7.60E-16 | 0.597 | -0.009 | 0.016 | 5.97E-01 | 1.04E-03 | 72 |
| 16 | 11924420 | rs10852344 | T | C | 0.590 | -0.160 | 0.020 | 1.30E-15 | 0.974 | -0.001 | 0.016 | 9.74E-01 | 9.22E-04 | 64 |
| 2 | 27569998 | rs704795 | A | G | 0.400 | -0.160 | 0.020 | 2.10E-15 | 0.125 | 0.025 | 0.016 | 1.25E-01 | 9.22E-04 | 64 |
| 6 | 31837338 | rs707938 | G | A | 0.320 | -0.170 | 0.020 | 7.20E-15 | 0.521 | 0.011 | 0.017 | 5.21E-01 | 1.04E-03 | 72 |
| 4 | 185985800 | rs6856693 | A | G | 0.580 | -0.160 | 0.020 | 9.80E-15 | 0.576 | 0.022 | 0.016 | 1.77E-01 | 9.22E-04 | 64 |
| 1 | 240084449 | rs2236918 | C | G | 0.450 | -0.150 | 0.020 | 8.30E-14 | 0.451 | -0.002 | 0.016 | 8.86E-01 | 8.10E-04 | 56 |
| 11 | 30183104 | rs11031006 | G | A | 0.850 | -0.220 | 0.030 | 8.50E-14 | 0.858 | 0.030 | 0.023 | 1.92E-01 | 7.75E-04 | 54 |
| 22 | 37209886 | rs763121 | G | A | 0.360 | -0.160 | 0.020 | 2.30E-13 | 0.436 | 0.013 | 0.017 | 4.36E-01 | 9.22E-04 | 64 |
| 19 | 61002040 | rs2547274 | G | C | 0.910 | -0.280 | 0.040 | 3.40E-13 | 0.905 | -0.043 | 0.027 | 1.19E-01 | 7.06E-04 | 49 |
| 6 | 11059723 | rs9393800 | G | A | 0.270 | -0.170 | 0.020 | 3.50E-13 | 0.402 | -0.015 | 0.018 | 4.02E-01 | 1.04E-03 | 72 |
| 5 | 175888783 | rs2241584 | A | G | 0.380 | -0.140 | 0.020 | 1.50E-11 | 0.992 | 0.000 | 0.016 | 9.92E-01 | 7.06E-04 | 49 |
| 2 | 47871585 | rs1800932 | A | G | 0.810 | -0.170 | 0.030 | 3.20E-11 | 0.805 | 0.036 | 0.020 | 7.52E-02 | 4.63E-04 | 32 |
| 12 | 129370287 | rs12824058 | G | A | 0.430 | -0.140 | 0.020 | 6.10E-11 | 0.661 | 0.007 | 0.016 | 6.61E-01 | 7.06E-04 | 49 |
| 17 | 38498992 | rs1799949 | G | A | 0.680 | -0.140 | 0.020 | 8.40E-11 | 0.677 | 0.017 | 0.017 | 3.15E-01 | 7.06E-04 | 49 |
| 15 | 39058739 | rs9796 | T | A | 0.460 | -0.130 | 0.020 | 1.30E-10 | 0.839 | -0.003 | 0.016 | 8.39E-01 | 6.09E-04 | 42 |
| 1 | 179228905 | rs1411478 | A | G | 0.410 | -0.130 | 0.020 | 1.40E-10 | 0.403 | -0.008 | 0.016 | 6.33E-01 | 6.09E-04 | 42 |
| 19 | 901694 | rs349306 | G | A | 0.130 | -0.230 | 0.040 | 1.70E-10 | 0.125 | 0.013 | 0.024 | 5.98E-01 | 4.76E-04 | 33 |
| 20 | 61019647 | rs13040088 | G | A | 0.210 | -0.160 | 0.020 | 2.40E-10 | 0.690 | -0.008 | 0.019 | 6.90E-01 | 9.22E-04 | 64 |
| 14 | 20003455 | rs1713460 | G | A | 0.300 | -0.140 | 0.020 | 2.40E-10 | 0.010 | 0.044 | 0.017 | 9.97E-03 | 7.06E-04 | 49 |
| 20 | 60760188 | rs2236553 | C | T | 0.240 | -0.160 | 0.030 | 6.10E-10 | 0.244 | -0.017 | 0.019 | 3.58E-01 | 4.10E-04 | 28 |
| 6 | 31633427 | rs2230365 | C | T | 0.840 | -0.170 | 0.030 | 7.60E-10 | 0.859 | 0.028 | 0.023 | 2.23E-01 | 4.63E-04 | 32 |
| 17 | 5272620 | rs8070740 | A | G | 0.760 | -0.150 | 0.020 | 1.50E-09 | 0.758 | -0.001 | 0.018 | 9.42E-01 | 8.10E-04 | 56 |
| 12 | 122166039 | rs1727326 | C | G | 0.150 | -0.190 | 0.030 | 1.70E-09 | 0.137 | -0.023 | 0.023 | 3.16E-01 | 5.78E-04 | 40 |
| 17 | 35086230 | rs2941505 | A | G | 0.320 | -0.130 | 0.020 | 1.90E-09 | 0.317 | -0.001 | 0.017 | 9.41E-01 | 6.09E-04 | 42 |
| 11 | 32498360 | rs10734411 | A | G | 0.470 | -0.120 | 0.020 | 2.60E-09 | 0.266 | 0.018 | 0.016 | 2.66E-01 | 5.19E-04 | 36 |
| 3 | 185106704 | rs16858210 | G | A | 0.750 | -0.140 | 0.020 | 3.10E-09 | 0.756 | -0.001 | 0.019 | 9.54E-01 | 7.06E-04 | 49 |
| 5 | 6798875 | rs427394 | G | A | 0.410 | -0.130 | 0.020 | 3.80E-09 | 0.063 | -0.030 | 0.016 | 6.34E-02 | 6.09E-04 | 42 |
| 8 | 61791955 | rs10957156 | A | G | 0.760 | -0.140 | 0.020 | 4.50E-09 | 0.236 | 0.022 | 0.018 | 2.36E-01 | 7.06E-04 | 49 |
| 20 | 5889999 | rs451417 | A | C | 0.120 | -0.200 | 0.030 | 4.60E-09 | 0.936 | -0.002 | 0.024 | 9.36E-01 | 6.40E-04 | 44 |
| 22 | 26963571 | rs5762534 | T | C | 0.840 | -0.160 | 0.030 | 6.10E-09 | 0.854 | 0.003 | 0.022 | 8.99E-01 | 4.10E-04 | 28 |
| 1 | 46519888 | rs12142240 | T | C | 0.680 | -0.130 | 0.020 | 6.60E-09 | 0.689 | -0.019 | 0.017 | 2.63E-01 | 6.09E-04 | 42 |
| 5 | 171867097 | rs11738223 | A | G | 0.680 | -0.120 | 0.020 | 2.00E-08 | 0.667 | -0.010 | 0.017 | 5.65E-01 | 5.19E-04 | 36 |
| 9 | 33002382 | rs4879656 | A | C | 0.370 | -0.120 | 0.020 | 2.00E-08 | 0.230 | -0.020 | 0.016 | 2.30E-01 | 5.19E-04 | 36 |
| 6 | 111704751 | rs12196873 | A | C | 0.850 | -0.160 | 0.030 | 2.80E-08 | 0.855 | -0.046 | 0.022 | 3.72E-02 | 4.10E-04 | 28 |
| 16 | 34355526 | rs12599106 | A | T | 0.510 | -0.120 | 0.020 | 3.10E-08 | 0.914 | 0.002 | 0.016 | 9.14E-01 | 5.19E-04 | 36 |
| 16 | 9112864 | rs9039 | C | T | 0.280 | -0.120 | 0.020 | 3.30E-08 | 0.257 | 0.020 | 0.018 | 2.57E-01 | 5.19E-04 | 36 |
| 12 | 119693576 | rs551087 | G | A | 0.290 | -0.130 | 0.020 | 3.90E-08 | 0.287 | -0.006 | 0.017 | 7.50E-01 | 6.09E-04 | 42 |
| 10 | 5809833 | rs10905065 | A | G | 0.610 | -0.110 | 0.020 | 3.90E-08 | 0.480 | -0.011 | 0.016 | 4.80E-01 | 4.36E-04 | 30 |
| 19 | 22299545 | rs7259376 | A | G | 0.460 | -0.110 | 0.020 | 4.20E-08 | 0.468 | -0.026 | 0.016 | 1.08E-01 | 4.36E-04 | 30 |
| 12 | 65100733 | rs7397861 | G | C | 0.640 | -0.100 | 0.020 | 6.70E-06 | 0.078 | 0.029 | 0.016 | 7.76E-02 | 3.60E-04 | 25 |
| 12 | 66698144 | rs2118138 | G | A | 0.520 | -0.090 | 0.020 | 1.90E-05 | 0.532 | 0.010 | 0.016 | 5.32E-01 | 2.92E-04 | 20 |
| 11 | 30263016 | rs6484478 | G | A | 0.740 | -0.100 | 0.020 | 4.00E-05 | 0.690 | 0.007 | 0.019 | 6.90E-01 | 3.60E-04 | 25 |
| 12 | 65021688 | rs1183272 | C | T | 0.450 | -0.070 | 0.020 | 0.00073 | 0.504 | -0.011 | 0.016 | 5.04E-01 | 1.77E-04 | 12 |

**Supplementary Table 4:** Detailed information for instrumental variables of age at first birth (AFB).

| **Chr** | **Position** | **rsID** | **Effect Allele** | **Other Allele** | **Exposure** | | | | **Outcome** | | | | **R^2^** | **F-stastic** |
| --- | --- | --- | --- | --- | --- | --- | --- | --- | --- | --- | --- | --- | --- | --- |
|  |  |  |  |  | **EAF** | **Beta** | **SE** | **p** | **EAF** | **Beta** | **SE** | **p** |  |  |
| 3 | 49898000 | rs2777888* | A | G | 0.507 | 0.106 | 0.013 | 4.58E-15 | 0.486 | -0.060 | 0.016 | 1.64E-04 | 2.65E-04 | 66 |
| 6 | 152229850 | rs2347867 | A | G | 0.649 | 0.091 | 0.015 | 1.38E-10 | 0.635 | -0.016 | 0.016 | 3.36E-01 | 1.47E-04 | 37 |
| 7 | 114313218 | rs10953766 | A | G | 0.429 | 0.087 | 0.014 | 1.82E-10 | 0.415 | -0.018 | 0.016 | 2.57E-01 | 1.54E-04 | 39 |
| 5 | 133898136 | rs10056247* | T | C | 0.289 | 0.082 | 0.016 | 2.32E-10 | 0.279 | 0.008 | 0.018 | 6.31E-01 | 1.05E-04 | 26 |
| 1 | 153927052 | rs10908557 | C | G | 0.695 | 0.091 | 0.015 | 5.59E-10 | 0.703 | -0.021 | 0.017 | 2.29E-01 | 1.47E-04 | 37 |
| 2 | 100832218 | rs1160544 | A | C | 0.395 | -0.082 | 0.014 | 2.90E-09 | 0.388 | -0.001 | 0.016 | 9.56E-01 | 1.37E-04 | 34 |
| 22 | 34107070 | rs242997 | A | G | 0.613 | -0.084 | 0.014 | 3.38E-09 | 0.605 | 0.019 | 0.016 | 2.27E-01 | 1.43E-04 | 36 |
| 20 | 31097877 | rs293566 | T | C | 0.650 | 0.081 | 0.015 | 1.41E-08 | 0.669 | -0.017 | 0.017 | 3.20E-01 | 1.16E-04 | 29 |
| 5 | 45094503 | rs6885307 | A | C | 0.799 | -0.107 | 0.017 | 4.37E-08 | 0.797 | 0.019 | 0.020 | 3.25E-01 | 1.58E-04 | 40 |
| 8 | 145677011 | rs2721195 | T | C | 0.469 | -0.073 | 0.016 | 6.25E-07 | 0.474 | 0.025 | 0.016 | 1.13E-01 | 8.29E-05 | 21 |

* : snp associated with BMI

**Supplementary Table 5:** Univariable MR results of hormonal reproductive on risk of OA.

| **Exposure/Outcome** | **Methods** | **No. of SNPs** | **OR (95%CI)** | ***P*** |
| --- | --- | --- | --- | --- |
| **AAM (11)** | | | | |
| Overall OA | Weighted median | 336 | 0.92(0.82-1.03) | 1.25E-01 |
| Overall OA | MR Egger | 336 | 0.96(0.79-1.13) | 6.35E-01 |
| Overall OA | Weighted mode | 336 | 0.92(0.69-1.15) | 4.56E-01 |
| Overall OA | Inverse variance weighted | 336 | 0.91(0.85-0.98) | 5.95E-03 |
| Hip OA | Weighted median | 337 | 1.13(0.93-1.33) | 2.18E-01 |
| Hip OA | MR Egger | 337 | 1.31(0.99-1.62) | 9.68E-02 |
| Hip OA | Weighted mode | 337 | 1.31(0.92-1.70) | 1.74E-01 |
| Hip OA | Inverse variance weighted | 337 | 1.01(0.89-1.13) | 8.56E-01 |
| Knee OA | Weighted median | 337 | 0.96(0.81-1.11) | 5.99E-01 |
| Knee OA | MR Egger | 337 | 0.97(0.72-1.22) | 8.06E-01 |
| Knee OA | Weighted mode | 337 | 1.07(0.79-1.36) | 6.22E-01 |
| Knee OA | Inverse variance weighted | 337 | 0.86(0.76-0.95) | 1.58E-03 |
| Hip and/or knee OA | Weighted median | 337 | 1.00(0.87-1.12) | 9.56E-01 |
| Hip and/or knee OA | MR Egger | 337 | 1.07(0.86-1.28) | 5.15E-01 |
| Hip and/or knee OA | Weighted mode | 337 | 1.18(0.89-1.47) | 2.72E-01 |
| Hip and/or knee OA | Inverse variance weighted | 337 | 0.92(0.84-1.00) | 3.64E-02 |
| **AAM (12)** | | | | |
| Overall OA | Weighted median | 119 | 0.93(0.79-1.06) | 2.53E-01 |
| Overall OA | MR Egger | 119 | 1.08(0.80-1.35) | 5.89E-01 |
| Overall OA | Weighted mode | 119 | 0.98(0.76-1.19) | 8.31E-01 |
| Overall OA | Inverse variance weighted | 119 | 0.94(0.86-1.03) | 2.09E-01 |
| Hip OA | Weighted median | 119 | 1.15(0.89-1.42) | 2.86E-01 |
| Hip OA | MR Egger | 119 | 1.63(1.07-2.20) | 9.20E-02 |
| Hip OA | Weighted mode | 119 | 1.17(0.74-1.60) | 4.84E-01 |
| Hip OA | Inverse variance weighted | 119 | 1.16(0.97-1.34) | 1.23E-01 |
| Knee OA | Weighted median | 119 | 1.00(0.80-1.20) | 9.96E-01 |
| Knee OA | MR Egger | 119 | 1.16(0.73-1.59) | 4.89E-01 |
| Knee OA | Weighted mode | 119 | 1.23(0.92-1.53) | 1.98E-01 |
| Knee OA | Inverse variance weighted | 119 | 1.02(0.85-1.19) | 2.31E-01 |
| Hip and/or knee OA | Weighted median | 119 | 1.33(0.97-1.68) | 8.31E-01 |
| Hip and/or knee OA | MR Egger | 119 | 1.12(0.86-1.38) | 1.20E-01 |
| Hip and/or knee OA | Weighted mode | 119 | 0.98(0.86-1.09) | 3.92E-01 |
| Hip and/or knee OA | Inverse variance weighted | 119 | 1.02(0.85-1.19) | 6.99E-01 |
| **Age at natural menopause (ANM)** | | | | |
| Overall OA | Weighted median | 51 | 1.00(0.96-1.05) | 9.05E-01 |
| Overall OA | MR Egger | 51 | 1.03(0.97-1.10) | 3.47E-01 |
| Overall OA | Weighted mode | 51 | 1.03(0.98-1.09) | 2.29E-01 |
| Overall OA | Inverse variance weighted | 51 | 1.00(0.97-1.03) | 9.05E-01 |
| Hip OA | Weighted median | 51 | 1.02(0.94-1.10) | 6.50E-01 |
| Hip OA | MR Egger | 51 | 1.07(0.94-1.19) | 3.18E-01 |
| Hip OA | Weighted mode | 51 | 1.09(0.98-1.20) | 1.34E-01 |
| Hip OA | Inverse variance weighted | 51 | 0.98(0.92-1.03) | 4.43E-01 |
| Knee OA | Weighted median | 51 | 0.98(0.92-1.05) | 5.80E-01 |
| Knee OA | MR Egger | 51 | 1.07(0.97-1.17) | 1.79E-01 |
| Knee OA | Weighted mode | 51 | 1.02(0.93-1.11) | 6.86E-01 |
| Knee OA | Inverse variance weighted | 51 | 1.00(0.96-1.04) | 9.46E-01 |
| Hip and/or knee OA | Weighted median | 51 | 1.03(0.97-1.08) | 3.33E-01 |
| Hip and/or knee OA | MR Egger | 51 | 1.07(1.00-1.15) | 6.77E-02 |
| Hip and/or knee OA | Weighted mode | 51 | 1.04(0.97-1.12) | 2.49E-01 |
| Hip and/or knee OA | Inverse variance weighted | 51 | 1.00(0.97-1.04) | 7.95E-01 |
| **Age at first birth (AFB)** | | | | |
| Overall OA | Weighted median | 10 | 0.81(0.65-0.97) | 1.02E-02 |
| Overall OA | MR Egger | 10 | 0.38(-0.65-1.40) | 9.85E-02 |
| Overall OA | Weighted mode | 10 | 0.81(0.56-1.07) | 1.50E-01 |
| Overall OA | Inverse variance weighted | 10 | 0.80(0.68-0.92) | 1.80E-04 |
| Hip OA | Weighted median | 10 | 0.73(0.39-1.08) | 7.61E-02 |
| Hip OA | MR Egger | 10 | 0.14(-1.97-2.25) | 1.06E-01 |
| Hip OA | Weighted mode | 10 | 0.58(0.03-1.13) | 8.52E-02 |
| Hip OA | Inverse variance weighted | 10 | 0.76(0.51-1.00) | 2.65E-02 |
| Knee OA | Weighted median | 10 | 0.80(0.57-1.04) | 6.54E-02 |
| Knee OA | MR Egger | 10 | 0.74(-0.81-2.28) | 7.07E-01 |
| Knee OA | Weighted mode | 10 | 0.77(0.42-1.13) | 1.88E-01 |
| Knee OA | Inverse variance weighted | 10 | 0.81(0.63-0.98) | 1.57E-02 |
| Hip and/or knee OA | Weighted median | 10 | 0.78(0.59-0.96) | 8.19E-03 |
| Hip and/or knee OA | MR Egger | 10 | 0.51(-0.76-1.78) | 3.31E-01 |
| Hip and/or knee OA | Weighted mode | 10 | 0.75(0.46-1.03) | 7.68E-02 |
| Hip and/or knee OA | Inverse variance weighted | 10 | 0.79(0.64-0.93) | 1.33E-03 |

MR: Mendelian randomization; OA: osteoarthritis; AAM: age at menarche; ANM: age at natural menopause; AFB: age at first birth; SNPs: single nucleotide polymorphisms

**Supplementary Table 6:** Univariable MR results of hormonal reproductive on risk of OA.

| **Exposure/Outcome** | **Methods** | **No. of SNPs** | **OR (95%CI)** | ***P*** |
| --- | --- | --- | --- | --- |
| **AAM (11) no BMI** | | | | |
| Overall OA | Weighted median | 285 | 0.96(0.86-1.06) | 4.15E-01 |
| Overall OA | MR Egger | 285 | 1.04(0.87-1.24) | 6.38E-01 |
| Overall OA | Weighted mode | 285 | 0.97(0.80-1.18) | 7.64E-01 |
| Overall OA | Inverse variance weighted | 285 | 0.96(0.90-1.03) | 3.01E-01 |
| Hip OA | Weighted median | 286 | 1.13(0.91-1.40) | 2.58E-01 |
| Hip OA | MR Egger | 286 | 1.49(1.07-2.07) | 1.99E-02 |
| Hip OA | Weighted mode | 286 | 1.31(0.90-1.90) | 1.54E-01 |
| Hip OA | Inverse variance weighted | 286 | 1.09(0.96-1.24) | 1.86E-01 |
| Knee OA | Weighted median | 286 | 1.15(0.89-1.48) | 2.78E-01 |
| Knee OA | MR Egger | 286 | 1.11(0.84-1.46) | 4.71E-01 |
| Knee OA | Weighted mode | 286 | 0.93(0.85-1.03) | 1.71E-01 |
| Knee OA | Inverse variance weighted | 286 | 1.01(0.88-1.16) | 8.90E-01 |
| Hip and/or knee OA | Weighted median | 286 | 1.22(0.98-1.52) | 7.13E-02 |
| Hip and/or knee OA | MR Egger | 286 | 1.19(0.92-1.54) | 1.81E-01 |
| Hip and/or knee OA | Weighted mode | 286 | 0.99(0.91-1.08) | 7.78E-01 |
| Hip and/or knee OA | Inverse variance weighted | 285 | 0.96(0.86-1.06) | 4.15E-01 |
| **AAM (12) no BMI** | | | | |
| Overall OA | Weighted median | 90 | 0.97(0.84-1.12) | 6.83E-01 |
| Overall OA | MR Egger | 90 | 1.05(0.81-1.38) | 7.02E-01 |
| Overall OA | Weighted mode | 90 | 1.02(0.82-1.26) | 8.87E-01 |
| Overall OA | Inverse variance weighted | 90 | 1.01(0.92-1.10) | 9.04E-01 |
| Hip OA | Weighted median | 90 | 1.20(0.90-1.60) | 2.25E-01 |
| Hip OA | MR Egger | 90 | 1.55(0.88-2.74) | 1.31E-01 |
| Hip OA | Weighted mode | 90 | 1.24(0.80-1.94) | 3.37E-01 |
| Hip OA | Inverse variance weighted | 90 | 1.23(1.01-1.49) | 4.28E-02 |
| Knee OA | Weighted median | 90 | 1.15(0.94-1.40) | 1.68E-01 |
| Knee OA | MR Egger | 90 | 1.16(0.78-1.73) | 4.66E-01 |
| Knee OA | Weighted mode | 90 | 1.30(0.94-1.80) | 1.18E-01 |
| Knee OA | Inverse variance weighted | 90 | 1.06(0.92-1.22) | 4.10E-01 |
| Hip and/or knee OA | Weighted median | 90 | 1.07(0.90-1.28) | 4.48E-01 |
| Hip and/or knee OA | MR Egger | 90 | 1.29(0.92-1.81) | 1.39E-01 |
| Hip and/or knee OA | Weighted mode | 90 | 1.15(0.87-1.52) | 3.30E-01 |
| Hip and/or knee OA | Inverse variance weighted | 90 | 1.09(0.97-1.22) | 1.60E-01 |
| **Age at first birth no BMI** | | | | |
| Overall OA | Weighted median | 8 | 0.81(0.69-0.96) | 1.40E-02 |
| Overall OA | MR Egger | 8 | 1.00(0.26-3.81) | 9.99E-01 |
| Overall OA | Weighted mode | 8 | 0.81(0.64-1.03) | 1.27E-01 |
| Overall OA | Inverse variance weighted | 8 | 0.83(0.72-0.94) | 4.91E-03 |
| Hip OA | Weighted median | 8 | 0.76(0.53-1.08) | 1.20E-01 |
| Hip OA | MR Egger | 8 | 0.29(0.02-4.56) | 4.13E-01 |
| Hip OA | Weighted mode | 8 | 0.70(0.39-1.25) | 2.69E-01 |
| Hip OA | Inverse variance weighted | 8 | 0.77(0.59-1.02) | 6.85E-02 |
| Knee OA | Weighted median | 8 | 0.82(0.64-1.06) | 1.34E-01 |
| Knee OA | MR Egger | 8 | 1.33(0.18-9.89) | 7.90E-01 |
| Knee OA | Weighted mode | 8 | 0.84(0.56-1.25) | 4.17E-01 |
| Knee OA | Inverse variance weighted | 8 | 0.80(0.65-0.98) | 2.83E-02 |
| Hip and/or knee OA | Weighted median | 8 | 0.78(0.63-0.95) | 1.60E-02 |
| Hip and/or knee OA | MR Egger | 8 | 0.89(0.17-4.69) | 9.00E-01 |
| Hip and/or knee OA | Weighted mode | 8 | 0.80(0.59-1.08) | 1.82E-01 |
| Hip and/or knee OA | Inverse variance weighted | 8 | 0.78(0.66-0.92) | 3.22E-03 |

MR: Mendelian randomization; OA: osteoarthritis; AAM: age at menarche; ANM: age at natural menopause; AFB: age at first birth; BMI: body mass index; SNPs: single nucleotide polymorphisms

**Supplementary Table 7.** Assessing directional pleiotropy through MR-Egger intercept and MR-PRESSO test among univariable MR.

| **Exposure/Outcome** | **MR-Egger intercept** | | |  | **MR-PRESSO** | |
| --- | --- | --- | --- | --- | --- | --- |
|  | **Intercept** | **SE** | **Pval** |  | **Global test Pval** | **Distortion test Pval** |
| **AAM (11)** | | | | | | |
| Overall OA | -0.002 | 0.003 | 0.539 |  | <0.001 | 0.772 |
| Hip OA | -0.010 | 0.006 | 0.085 |  | 0.004 | 0.878 |
| Knee OA | -0.005 | 0.005 | 0.301 |  | <0.001 | 0.212 |
| Hip and/or knee OA | -0.006 | 0.004 | 0.118 |  | <0.001 | NA |
| **AAM (12)** | | | | | | |
| Overall OA | -0.006 | 0.006 | 0.318 |  | 0.001 | 0.779 |
| Hip OA | -0.016 | 0.013 | 0.208 |  | <0.001 | 0.808 |
| Knee OA | -0.011 | 0.010 | 0.255 |  | <0.001 | NA |
| Hip and/or knee OA | -0.014 | 0.008 | 0.076 |  | <0.001 | NA |
| **ANM** | | | | | | |
| Overall OA | -0.006 | 0.006 | 0.323 |  | 0.210 | NA |
| Hip OA | -0.017 | 0.012 | 0.139 |  | 0.922 | NA |
| Knee OA | -0.014 | 0.009 | 0.128 |  | 0.204 | NA |
| Hip and/or knee OA | -0.014 | 0.007 | 0.057 |  | 0.567 | NA |
| **AFB** | | | | | | |
| Overall OA | 0.067 | 0.046 | 0.187 |  | 0.375 | NA |
| Hip OA | 0.150 | 0.095 | 0.155 |  | 0.448 | NA |
| Knee OA | 0.008 | 0.070 | 0.911 |  | 0.861 | NA |
| Hip and/or knee OA | 0.039 | 0.057 | 0.520 |  | 0.849 | NA |

NA, no outliers were detected. MR: Mendelian randomization; OA: osteoarthritis; AAM: age at menarche; ANM: age at natural menopause; AFB: age at first birth; BMI: body mass index

**Supplementary Figures**

**
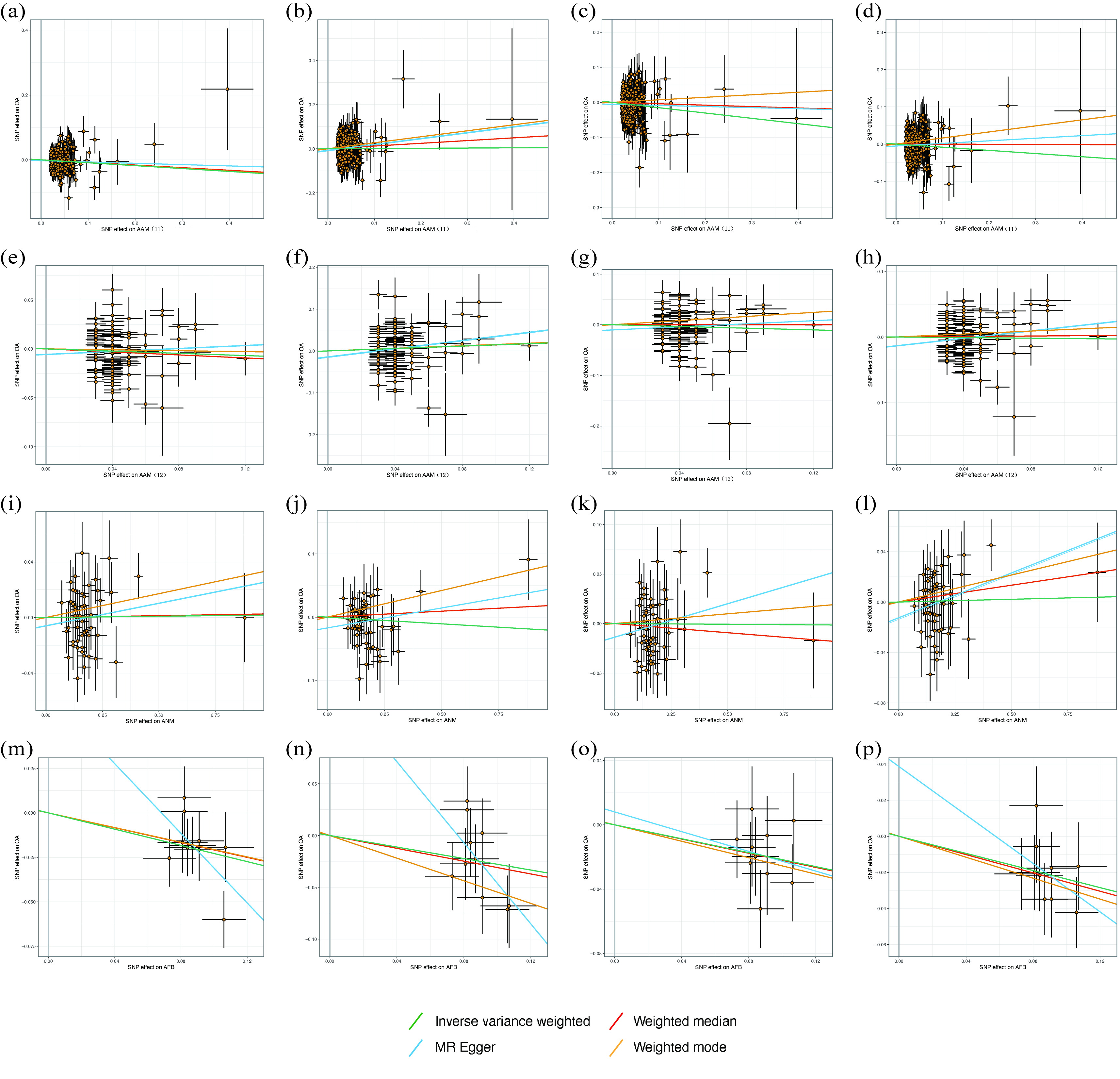
**

**Supplementary Fig. 1:** **Scatter plots for effect sizes of SNPs for hormonal reproductive factors and those for OA and subtypes.** (a-d) AAM (11); (e-h) AAM (12); (i-l) ANM; and (m-p) AFB. The x-axis represents the effect size of SNPs on hormonal reproductive factors; the y-axis represents the effect size of SNPs on osteoarthritis and subtypes. Colors of fitted line represents for four approaches used in univariable MR analyses. MR: Mendelian randomization; OA: osteoarthritis; AAM: age at menarche; ANM: age at natural menopause; AFB: age at first birth; SNPs: single nucleotide polymorphisms


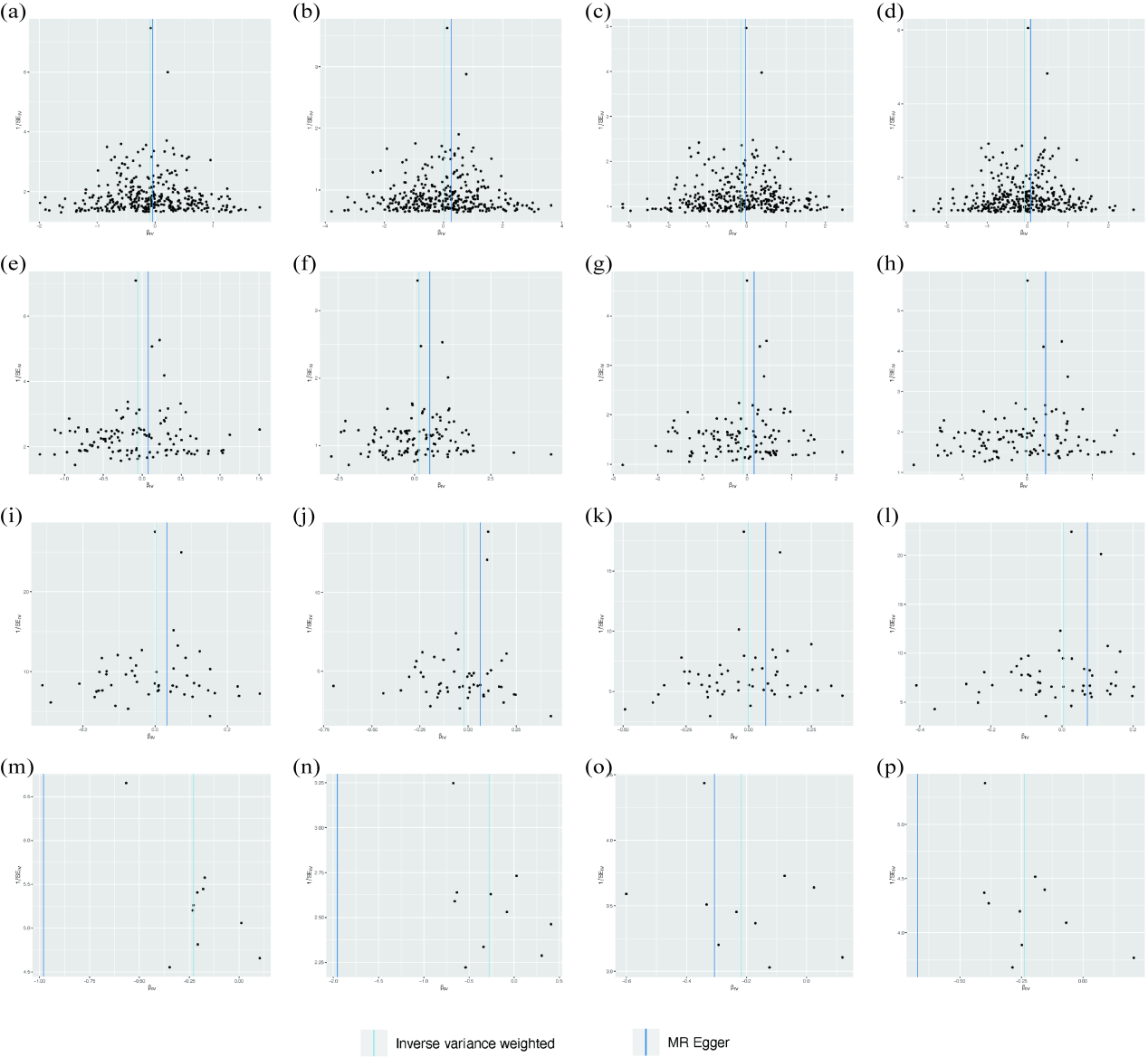


**Supplementary Fig. 2: Funnel plots to show symmetrical distribution of individual variant estimates around the point estimate.** (a-d) AAM (11); (e-h) AAM (12); (i-l) ANM; and (m-p) AFB. The x-axis represents the MR estimate of individual SNPs; the y-axis represents the inverse of their standard error. MR: Mendelian randomization; OA: osteoarthritis; AAM: age at menarche; ANM: age at natural menopause; AFB: age at first birth; SNPs: single nucleotide polymorphisms
